# Supplementary material for: Validity and reliability of the Swedish Functional Health Literacy scale and the Swedish Communicative and Critical Health Literacy scale in patients undergoing bariatric surgery in Sweden: a prospective psychometric evaluation study
Source: BMJ Open. 2021 Nov 29;11(11):e056592. doi: 10.1136/bmjopen-2021-056592 (PMC8634233; doi:10.1136/bmjopen-2021-056592)
Supplement: Author's manuscript [file bmjopen-2021-056592.draft_revisions.pdf]

BMJ Open is committed to open peer review. As part of this commitment we make the peer review history of every article we publish publicly available.

When an article is published we post the peer reviewers' comments and the authors' responses online. We also post the versions of the paper that were used during peer review. These are the versions that the peer review comments apply to.

The versions of the paper that follow are the versions that were submitted during the peer review process. They are not the versions of record or the final published versions. They should not be cited or distributed as the published version of this manuscript.

BMJ Open is an open access journal and the full, final, typeset and author-corrected version of record of the manuscript is available on our site with no access controls, subscription charges or pay-per-view fees (http://bmjopen.bmj.com).

If you have any questions on BMJ Open's open peer review process please email info.bmjopen@bmj.com

BMJ Open

The Swedish Functional Health Literacy scale and the Swedish Communicative and Critical Health Literacy scale in patients undergoing bariatric surgery: a prospective psychometric evaluation study

Journal:	*BMJ Open*
Manuscript ID	bmjopen-2021-056592
Article Type:	Original research
Date Submitted by the Author:	24-Aug-2021
Complete List of Authors:	Jaensson, Maria; Örebro University, Faculty of Medicine and Health, School of Health Sciences<br>Stenberg, Erik; Örebro universitet Fakulteten för medicin och hälsa, Faculty of Medicine and Health<br>Liang, Yuli; Örebro University School of Business, Department of Statistics<br>Nilsson, Ulrica; Karolinska Institutet, Department of Neurobiology, Care Sciences and Society, Karolinska Institutet Perioperative Medicine and Intensive Care; Karolinska Universitetssjukhuset, Perioperative Medicine and Intensive Care<br>Dahlberg, Karuna; Örebro University, Faculty of Medicine and Health, School of Health Sciences
Keywords:	Adult surgery < SURGERY, Health & safety < HEALTH SERVICES ADMINISTRATION & MANAGEMENT, STATISTICS & RESEARCH METHODS

SCHOLARONE™
Manuscripts

BMJ

I, the Submitting Author has the right to grant and does grant on behalf of all authors of the Work (as defined in the below author licence), an exclusive licence and/or a non-exclusive licence for contributions from authors who are: i) UK Crown employees; ii) where BMJ has agreed a CC-BY licence shall apply, and/or iii) in accordance with the terms applicable for US Federal Government officers or employees acting as part of their official duties; on a worldwide, perpetual, irrevocable, royalty-free basis to BMJ Publishing Group Ltd ("BMJ") its licensees and where the relevant Journal is co-owned by BMJ to the co-owners of the Journal, to publish the Work in this journal and any other BMJ products and to exploit all rights, as set out in our licence.

The Submitting Author accepts and understands that any supply made under these terms is made by BMJ to the Submitting Author unless you are acting as an employee on behalf of your employer or a postgraduate student of an affiliated institution which is paying any applicable article publishing charge ("APC") for Open Access articles. Where the Submitting Author wishes to make the Work available on an Open Access basis (and intends to pay the relevant APC), the terms of reuse of such Open Access shall be governed by a Creative Commons licence – details of these licences and which Creative Commons licence will apply to this Work are set out in our licence referred to above.

Other than as permitted in any relevant BMJ Author's Self Archiving Policies, I confirm this Work has not been accepted for publication elsewhere, is not being considered for publication elsewhere and does not duplicate material already published. I confirm all authors consent to publication of this Work and authorise the granting of this licence.

For peer review only

The Swedish Functional Health Literacy scale and the Swedish Communicative and Critical Health Literacy scale in patients undergoing bariatric surgery: a prospective psychometric evaluation study

Jaensson M[1], Stenberg E [2], Liang, Y[4], Nilsson U[3], Dahlberg K [1]

1. Faculty of Medicine and Health. School of Health Sciences, Örebro University, Örebro, Sweden
2. Department of Surgery, Faculty of Medicine and Health, Örebro University, Örebro, Sweden
3. Department of Neurobiology, Care Sciences, and Society, Karolinska Institutet and Perioperative Medicine, Karolinska University Hospital, Stockholm, Sweden
4. Department of Statistics, Örebro University School of Business, Örebro, Sweden

Corresponding author: Maria Jaensson, School of Health Sciences, Örebro University, Fakultetsgatan 1, SE- 701 82,ÖREBRO, Sweden
maria.jaensson@oru.se

Word count:3499

For peer review only

ABSTRACT

Objectives

The aim was to psychometrically test and evaluate the Swedish functional health literacy scale and the Swedish communicative and critical health literacy scale in patients undergoing bariatric surgery.

Design

A prospective cross-sectional psychometric study

Setting

Patients from three bariatric centers in Sweden were consecutively included in this study.

Participants

A total of 704 patients undergoing bariatric surgery filled in the questionnaires preoperatively. Inclusion criteria were scheduled for primary bariatric surgery (Roux-en-Y gastric bypass or sleeve gastrectomy) and greater than 17 years, proficiency in Swedish.

Primary and secondary measures

Psychometric outcomes of the Swedish functional health literacy scale and the Swedish communicative and critical health literacy scale

Results

There was a higher proportion of females (74.4%, n=523) to males (25.6%, n=180). The mean age was 42 years (SD 11.5). Limited functional health literacy and limited communicative and critical health literacy (including both inadequate and problematic health literacy) was reported in 55 % (n=390) and 40 % (n=285), respectively. Cronbach alpha for the Swedish functional health literacy scale was α= 0.86 and for the Swedish communicative and critical health literacy scale, α=0.87. Construct validity showed weak to negative correlations between the Swedish functional health literacy scale and income, education, and SF-36/RAND36 summary scores. Confirmatory factor analysis showed a one-factor solution for the Swedish functional health literacy scale and a two-factor solution for the Swedish communicative and critical health literacy scale.

Conclusions

The Swedish functional health literacy scale and the Swedish communicative and critical health literacy scale are valid and reliable to use for patients undergoing bariatric surgery in a Swedish context. Measuring dimensions of health literacy can be used as a guide for the development of health literacy friendly patient information in patients undergoing bariatric surgery.

For peer review only

ARTICLE SUMMARY

Strengths and limitations of this study

- This is the first psychometric evaluation in a Swedish sample undergoing bariatric surgery.
- This study contributes to the development of valid and reliable scales to be used in a clinical context.
- This study shows high prevalence of limited health literacy in patients undergoing bariatric surgery.
- The timing of preoperative data collection can be a limitation.
- There are few previous psychometric evaluations, making it difficult to compare results.

For peer review only

For peer review only - http://bmjopen.bmj.com/site/about/guidelines.xhtml

INTRODUCTION

The concept of health literacy has been known for more than 3 decades.[1] Health literacy can be defined as "*a person's ability to assess, understand and use information to maintain or improve one´s health*".[2] Health literacy can be divided into different skills or dimensions. Functional health literacy covers basic skills in understanding and using health information. Communicative and critical health literacy refers to more advanced social and cognitive skills in communication, applying and discriminating between different sources of information.[3, 4] In recent years, research has investigated the consequences of limited health literacy for both the individual and the health care system. Factors such as lower educational level, lower household income, refugees, older age, and sex are associated with limited health literacy.[5-10] Limited health literacy has been reported to be associated with poorer general health, poorer postoperative recovery and increased health care cost.[10-15] For the patient with limited health literacy, it can be difficult to adhere to a medication regimen and to understand health information.[10] Of particular note is research that has shown that a patient with limited health literacy may ask fewer questions when meeting and talking to health care providers.[16] Knowledge of health literacy is therefore important for health care providers to provide equal care for all patients, especially when advocating a person-centered approach.[17]

Scales and instruments can measure health literacy, but there is no scale or instrument that is considered the gold standard.[18] This study uses two different scales measuring functional health literacy, namely the Swedish Functional Health Literacy (FHL) scale and the Swedish Communicative and Critical Health Literacy (C & C HL) scale. Both scales were originally developed and revised in Japan and have previously been translated to Swedish and are psychometrically tested and found to be valid and reliable in a Swedish context.[19-22] Exploratory factor analysis by Ishikawa et al. supported a one-factor model for both scales.[19, 23] The scales' psychometric properties have been evaluated in different populations, for instance, general population, patients with diabetes, refugees, patients undergoing day surgery, and outpatient clinics attendees.[7, 8, 11, 19, 21, 22]

The prevalence of limited health literacy in a variety of surgical patients has been shown in a meta-analysis to be greater than a third (34%, interquartile range 16%-50%).[18] Previous research, focusing on patients undergoing bariatric surgery, shows that 30-50 % of this population has limited health literacy.[24-26] Bariatric surgery leads to a decrease in morbidity and mortality due to obesity and is the most effective method to reduce weight long term.[27, 28] This surgery requires patients to adhere to several health care promotion guidelines to minimize surgical complications, loose excessive weight, and learn healthier behavior.[29] Therefore patients are carefully prepared before surgery with education, among other things. However, analysis of patients' recall of information shows much information is forgotten or may not have been understood.[30] There is a risk, therefore, that patients with limited health literacy cannot fully adhere to desired health care promotion guidelines pre- and postoperatively with weight loss surgery.

When investigating a phenomenon such as health literacy, it is of utmost importance that the scale or instrument is psychometrically evaluated when used in different contexts, recognizing

For peer review only - http://bmjopen.bmj.com/site/about/guidelines.xhtml

that scales or instruments can be used to design patient information or education or to facilitate communication between health care provider and patient.[3, 31] This study therefore aims to psychometrically test and evaluate the Swedish FHL scale, and the Swedish C & C HL scale in patients undergoing bariatric surgery.

METHOD

Study design

This psychometric evaluation was a cross-sectional survey conducted between October 2018 and November 2020 consisting of preoperative paper-based self-reported functional and communicative and critical health literacy in patients undergoing bariatric surgery. This study is part of a multicentre prospective, longitudinal mixed-methods study with an embedded design.[32]

Ethics

The project was approved by the Regional Ethical Review Board in Uppsala, Sweden (ref: 2018/256) and followed good clinical practice and ethical principles as outlined by the 1964 Helsinki Declaration and its later amendments. All participants received written and oral information, including voluntary written consent and the option to withdraw at any time.

Sample and settings

Patients from three centers in Sweden were consecutively included in this study. The inclusion criteria were planned primary bariatric surgery (Roux-en-Y gastric bypass or sleeve gastrectomy) and greater than17 years proficiency in Swedish.

All centers performing bariatric surgical procedures in Sweden report to the national quality and research register (Scandinavian Obesity Surgery Registry [SOReg]).[33] Data in the SOReg are collected at different timepoints: baseline (pre- and intraoperatively) and up to 10 years after surgery. For this psychometric study, cross sectional data has been merged with data from SOReg collected at baseline before surgery.

Information about this study was given by a surgeon (in one center) and a trained research nurse (in two centers) on a preoperative visit. If the patient agreed to participate, informed written consent was collected on the day of the surgery before filling in the study-specific form.

Patient and Public Involvement

No patient involved.

Data collection

To measure health literacy, the Swedish FHL scale and the Swedish C & C HL scale were used. Both scales consist of 5 items each. The Swedish FHL scale's first two items focus on visual ability related to the design of the text and its accessibility, the next two on the understanding of words and concepts, the fourth on perseverance in reading, and the last on

For peer review only - http://bmjopen.bmj.com/site/about/guidelines.xhtml

the need for help in reading and understanding information. Responses are on a 5-point ordinal scale (1=never to 5=always) with lower scores indicating sufficient health literacy. [4]

The first three items in the Swedish C & C HL scale focus on the capacity for collecting, extracting, and understanding information related to health. The last two items focus on the abilities required to judge the reliability of the information and to apply health information to everyday life. Responses are on a 5-point ordinal scale (1=strongly disagree to 5=strongly agree), with higher scores indicating a sufficient health literacy. [21, 22]

Neither scale provides an initial summary score; instead, reported variables are re-categorized to three different levels (inadequate, problematic or sufficient health literacy)[4, 34]

To evaluate construct and discriminant validity, the following baseline variables were collected from SOReg: patient characteristics (age, gender, weight, BMI and type of surgery) and data from two additional scales, both of which have been found to be valid and reliable for Swedish contexts.[35-37] The first, SF-36/RAND36 (hereafter referred to simply as SF-36), measures health-related quality of life and consists of 36 items grouped into eight scales (bodily pain, emotional role, general health, mental health, physical function, role function, social functioning, and vitality). A higher score indicates better health status (ranging from 0 to 100). SF-36 provides two summary scores: the physical component summary (PCS) and the mental component summary (MCS) reflecting overall physical and mental health status. [35, 36] The second, the obesity-related problem (OP)scale measures the impact of obesity on psychosocial functioning. It consists of eight questions on common obesity-related problems. A low score indicates better psychosocial functioning (ranging from 0 to 100).[37]

Psychometric testing and statistical analysis `

The psychometric evaluation was guided by the Consensus-Based Standards for the Selection of Health Measurement Instruments (COSMIN) guidelines.[38] There was no priori sample size calculation for this psychometric evaluation study.

Internal consistency measures the degree of interrelatedness and Cronbach alpha; coefficients ≥0. 70 and ≤0.95 were regarded as acceptable. [39, 40] Inter-item correlation was analyzed to indicate if an item was part of the scale; appropriate values were 0.2 to 0.50.[41]

Floor and ceiling effects for each item were calculated as percentages of ratings at the lowest or highest level and was taken into account if more than 15% reported the lowest or the highest score.[40]

Construct validity was tested with predefined hypotheses drawn from previous research, namely that those participants with limited functional health literacy have significantly lower educational level, lower income, and lower preoperative summary scores in PCS and MCS in SF-36. [9, 10] When analyzing correlations, the Swedish FHL scale and Swedish C & C HL scale were the dependent variables. To investigate differences between groups (health literacy and educational level and income), the Chi-square-test was used. Correlations were analyzed using the Spearman *rho* correlation test and were interpreted as follows: < 0.10 was considered a negligible correlation, 0.10-0.39 week correlations, 0.40-0.69 moderate correlations, 0.70-0.89 strong correlations, and >0.89 very strong correlations. [42]

For peer review only - http://bmjopen.bmj.com/site/about/guidelines.xhtml

Structural validity was tested with confirmatory factor analysis (CFA).[38] We assumed a 1-factor model with 5 items for the Swedish FHL scale and a 2-factor model for the Swedish C & C HL scale.[43] Before running the CFA, the presence of outliers was investigated. Based on the squared Mahalanobis distance, there were 28 outliers and 29 outliers detected in the questionnaires of Swedish FHL and Swedish C&C HL, respectively. The CFA was conducted after removing the outlier observations. The following model fit criteria were used to evaluate the fit of the hypothetical model comparative fit index (CFI) > 0.90 [44], root mean square error of approximation (RMSEA) < 0.08 [45] and standardized root mean square residual (SRMR) < 0.08.[46]

Discriminant validity was tested with the a priori hypotheses that the OP-scale has weak association (r=≤0.39) with both the Swedish FHL and the Swedish C & C HL scale.[42]

Sociodemographic variables and health literacy were analyzed descriptively and presented as number, percent, mean and standard deviation (SD).

Functional health literacy was categorized manually into sufficient, problematic and inadequate by re-categorization using the following transformations: 1and 2 became 1; 3 became 100; and 4 and 5 became 1000. All values were summed and answers >1000 interpreted as inadequate, >100 and <1000 problematic, and <100 sufficient functional health literacy. [4] Communicative and critical health literacy was categorized into sufficient, problematic and inadequate by re-categorization as follows; 1and 2 became 1000; 3 became 100;, and 4 and 5 became 1. All values were summed and totals ≥1000 interpreted as inadequate, >100 and <1000 as problematic, and <100 as sufficient communicative and critical health literacy. [34] Health literacy levels (both scales) were further dichotomized into limited (including inadequate/problematic) vs. sufficient. Missing data was handled by listwise deletion.

The self-reported educational level was mapped to low and high educational level (compulsory school, upper secondary school, and higher vocational training vs. first and second cycle education and third cycle study programs) [47]. Higher vocational training in Sweden lasts from one to two years [48]. The self-reported variable of income was mapped to low and high income (cut-off of 300 000 Swedish krona [SEK]/year, where 1 EUR equals approximately 10.8 SEK [June 2021]) and it was guided by the Statistics Sweden website which reports a median income of 337 000 SEK/year (2019) for men and women (20-64 year) [49].

A p<0.05 value was considered significant. IBM SPSS Statistics, version 26, was used for the calculations. CFA was performed using the lavaan package, version 0.6-3. [50]

RESULTS

A total of 704 patients gave their consent to participate in the study. Six forms were unit nonresponses and 3 forms had partial responses in the Swedish FHL scale. Seven forms were unit nonresponses and 16 forms had partial responses in the Swedish C & C HL. Missing answers varied between 6 and 16 in each item in both scales. Most missing answers were in

For peer review only - http://bmjopen.bmj.com/site/about/guidelines.xhtml

item 4 in Swedish C & C HL scale, "*Considering the credibility of the information.*". Response rate for the different scales or instruments were 98.7% (n= 695/704) for the Swedish FHL scale, 96.7% (n=681/704) for the Swedish C & C HL scale, and 72.3% (n=509/704) for the SF-36 and OP scales.

There was a higher proportion of females, 74.4% (n=523) to 25.6% (n=180) males. The mean age was 42 years (SD 11.5), and the mean preoperative weight and BMI were 120.2 kilogram (SD 21.9) and 41.7 (SD 5.7) respectively. In this sample, 55% (n=390) reported limited FHL (including both inadequate and problematic functional health literacy) and 40% (n=285) had limited C & C HL. Most patients reported upper secondary school as their highest level of education (52%, n=367/n=698). Almost twenty-five percent (24.6%) had undergone first, second or third cycle programmes at universities. The most frequent type of surgery was gastric bypass (56.8%, n=400) (Table 1).

Table 1. Sociodemographic variables, health literacy results, and type of surgery

**Variables**	
Sex Female/Male n (%)	523(74.4)/180(25.6)
Age years M(SD)	41.77 (11.4)
Weight (kg) M(SD)[a]	120.27 (21.9)
BMI M(SD)[a]	41.77 (5.7)
**Level of educational n (%)**	
Compulsory school	67 (9.6)
Upper secondary school	367 (52.6)
Other education (*i.e.*, higher vocational training)	92 (13.2)
First and second cycle study programmes	171 (24.5)
Third cycle study programmes	1 (0.1)
**Income (SEK[b]) n (%)**	
<200 000	63 (9.1)
200 000-300 000	97 (13.9)
300 000-400 000	135 (19.4)
400 000-500 000	108 (15.3)
500 000-600 000	89 (12.8)
>600 000	167 (24)
Unknown	37 (5.3)
**Swedish functional health literacy (FHL) n (%)**	
Inadequate	115(16)
Problematic	275(39)
Sufficient	305(43)
**Swedish communicative and critical health literacy (C & C HL) n (%)**	
Inadequate	45(6)
Problematic	240(34)
Sufficient	395(56)

For peer review only - http://bmjopen.bmj.com/site/about/guidelines.xhtml

**Type of surgery** n (%)	
Gastric bypass (GBP)	400 (56.8)
Sleeve gastrectomy (SG)	292 (41.5)
Missing	10
Other	2 (0.2)

[a]n=698, [b]SEK=Swedish kronor, M-mean, SD-standard deviation

Item content and mean (SD) scores for the Swedish FHL scale and the Swedish C & C HL scale are seen in Table 2.

Floor ceiling effect/response distribution

There was a floor and ceiling for all items in both scales (Table 2). The Swedish FHL scale had a floor effect with answers skewed to the left and between 24.6 and 61.4% of the answers to each item being "never." For the Swedish C & C HL scale answers were skewed to the right and questions were answered "correct". The ceiling effect varied between 27.6 and 66.9% for all items (Table 2).

Internal consistency

Cronbach alpha for the Swedish FHL scale was α= 0.86 and for the Swedish C & C HL scale, α=0.87. Inter-item correlations varied between r=0.444 and r=0.702 (Swedish FHL scale) and r=0.434 and r=0.702 (Swedish C & C HL scale).

For peer review only

For peer review only - http://bmjopen.bmj.com/site/about/guidelines.xhtml

Table 2. Item content, response distributions for each item, and mean (SD) and inter-item correlations for the Swedish FHL and the Swedish C & C HL scales

**Swedish functional health literacy (FHL)[a]**	**n**	**n (%) missing**	**Response category n (%)**					**Mean (SD)**
			**1**	**2**	**3**	**4**	**5**	
1. Do you think that it is difficult to read health information because the text is difficult to see (even if you have glasses or contact lenses)?	697	7(1)	349(49.6)	187(26.6)	131(18.6)	25(3.6)	5(0.7)	1.78(0.92)
2. Do you think that it is difficult to understand word or numbers in health information?	697	7(1)	269(38.2)	231(32.8)	164(23.3)	28(4.0)	5(0.7)	1.95(0.91)
3. Do you think that it is difficult to understand the message in health information?	697	7(1)	263(37.4)	258(36.6)	153(21.7)	20(2.8)	3(0.4)	1.91(0.86)
4. Do you think that it takes a long time to read health information?	698	6(0.9)	173(24.6)	216(30.7)	234(33.2)	57(8.1)	18(2.6)	2.33(1.01)
5. Do you ever ask someone else to read and explain health information?	698	6(0.9)	432(61.4)	150(21.3)	76(10.8)	32(4.5)	8(1.1)	1.61(0.93)
**Swedish communicative and critical health literacy (C & C HL)[b]**	**n**	**n (%) missing**	**Response category n (%)**					**Mean (SD)**
			**1**	**2**	**3**	**4**	**5**	
1. Seeking information from various) sources	696	8(1.1)	2(0.3)	4(0.6)	36(5.1)	183(26)	471(66.9)	4.61 (0.63)
2. Extracting relevant information	696	8(1.1)	4(0.6)	17(2.4)	84(11.9)	259(36.8)	332(47.2)	4.29(0.81)
3. Understanding and communicating the information	695	9(1.3)	3(0.4)	7(1.0)	112(15.9)	285(40.5)	288(40.9)	4.23(0.76)
4. Considering the credibility of the information	688	16(2.3)	5(0.7)	19(2.7)	178(25.3)	292(41.5)	194(27.6)	3.95(0.84)
5. Making decisions based on the information	693	11(1.6)	4(0.6)	6(0.9)	121(17.2)	320(45.5)	242(34.4)	4.14(0.77)

Swedish functional health literacy (FHL) scale: 1= never, 2=rarely, 3=sometimes, 4=often, 5=always. Swedish communicative and critical (C & C HL) scale: 1= not correct at all, 2=incorrect, 3=partly true, 4= correct, 5=exactly right. [a]English translation of items by Wångdahl et al.[4]
[b]English translation of items by Ishikawa et al.[19]

For peer review only - http://bmjopen.bmj.com/site/about/guidelines.xhtml

Construct validity

Bivariate analyses showed significant associations with limited functional health literacy for the independent variables of income (p=0.01) and educational level (p= 0.02). Limited FHL was associated with lower income and lower educational level. There were negligible to weak negative correlations in all four a priori hypotheses (income, education, MCS and PCS) (Table 3). There was no significant association between education and income with communicative and critical health literacy. When analyzing discriminant validity there was a negligible association between the OP scale and the Swedish FHL scale (*rho* 0.054, p= 0.22) and the C & C HL scale (*rho* 0.046, p= 0.31).

Table 3. Spearman *rho* correlations between the Swedish functional health literacy (FHL) scale and both the demographic characteristics and SF 36

**Variable**	**Value,** *rho*	***P* value**
Education level	-.122**	**0.003**
Income	-.097*	**0.01**
SF-36 MCS	-.251**	**<0.001**
SF-36, PCS	-.103	**0.021**

*Correlation is significant at the 0.05 level (two-tailed),

** Correlation is significant at the 0.001 level (two-tailed)

SF-36, MCS=mental component score and PCS=physical component score

Confirmatory factor analysis

The global fit can be assessed by the following criteria: CFI=0.996, RMSEA=0.043 and SRMR=0.013. The model shown in Figure 1 had a very good model fit which indicates that the one-factor solution is a nice fitting model of the Swedish FHL scale in patients undergoing bariatric surgery. Figure 1 shows the path diagram of the Swedish FHL as well as the factor loadings estimated by the maximum likelihood estimation method. The factor FHL explained almost half or more than half of the variances of items a, d, and e (0.46-0.63 with p-values < 0.001) while less than one-third of the variances of items b and c were explained by the factor FHL (0.27 and 0.33 with p-values < 0.001). The factor loadings, which measured the correlations between a specific item and the FHL factor, ranged from 0.61 to 0.85 (p-values < 0.001).

Insert Figure 1

CFA showed a good fit for a two-factor model with correlation between 2 factors given in Figure 2: CFI = 0.996, RMSEA = 0.047 (90% confidence interval 0.007-0.085) and SRMR = 0.012. The value 0.88 on the curved double-headed arrows is the correlation between the two factors. Comparing this 2-correlated-factor CFA model with the one-factor solution shows that the model in Figure 2 has a better fit.

Insert Figure 2

For peer review only - http://bmjopen.bmj.com/site/about/guidelines.xhtml

DISCUSSION

This study shows that the Swedish FHL scale and the Swedish C & C HL scale are valid and reliable to use in patients undergoing bariatric surgery.

This study represents a sufficient sample size according to COSMIN guidelines [38] and also has high response rates with 695/704 (Swedish FHL scale) and 681/704 (Swedish C & C HL scale) responses.

When analyzing the patients' responses, it was evident that both scales showed a skewed response distribution. This resulted from the large proportion of patients that reported answers on either the lower end (Swedish FHL scale) or the higher end (Swedish C & C HL scale) of the items. As far as we know, this response pattern has not been analysed in previous studies. The reason for this result in our study is not clear. It might be a characteristic of patients undergoing bariatric surgery. It might be a drawback of the construction of both scales.[51] However, the response scales in both the Swedish FHL scale and the Swedish C & C HL scale are categorical rating scales not different from other instruments measuring health literacy. For example, the HLS-EU-Q47 and the HL-EU Q16 have similar 4-point Likert scales with response options on a 4-point scale ranging from 'very difficult' to 'very easy. [52]

One item stood out with the most missing answers (n=16), item 4 in the Swedish C & C HL scale, "*Considering the credibility of the information*". The reason for this is not clear though we did not perform a face or content validity assessment. One explanation might be that the patients found it difficult to value their own skill in knowing if information is credible or not. Responses to specific items are not to be found in previous psychometric evaluations. [20, 22, 23], suggesting that they should be studied further to see if it is an issue in other contexts as well.

Our analysis showed that both scales has good reliability with acceptable Cronbach alpha values of 0.86 (Swedish FHL scale) and 0.87 (Swedish C & C HL scale).[41] This internal consistency is in line with recommended values.[39, 40] Our result is also consistent with previous research on the FHL scale (Cronbach alpha of 0.684) and on the C & C HL (Cronbach alpha 0.86).[19, 23] In addition, inter-item correlations showed acceptable values, although one value of 0.702 indicates that the two items measure almost the same thing, with high inter-item correlation values possibly showing that some items in the scales may be redundant. [41]
Construct validity was confirmed for the Swedish FHL scale by showing an association, although weak to negligible, with income, educational level, and overall poorer health. [9, 25, 53] When interpreting these data, it is important to bear in mind that correlation coefficient association strengths are often interpreted literally, a misinterpretation that is problematic because associations do not show causal relationships.[42] Our results contribute to the growing body of evidence surrounding health literacy's impact on the individual as well as on the health care system.[3]

For peer review only - http://bmjopen.bmj.com/site/about/guidelines.xhtml

When applying CFA to investigate the factor structures for the scales, we obtained a 1-factor model for FHL and a 2-factor model for C & C HL. One item (CC3) "*understanding and communicating the information*" is now loaded on the critical dimension instead of the communicative dimension. This loading might be explained by the wording of this item, because "understand" refers more strongly to critical aspects and people often share information with others after some critical analysis. Moreover, a better model fit was observed when CC3 was included in the critical dimension rather than the communicative dimension. Previous studies have also considered communicative and critical dimensions are two processing dimensions.[3, 54]

The concept of health literacy has been known for decades and its importance to equality acknowledged.[55] Extensive research has shown challenges with limited health literacy not only for the individual but also for health care professionals. Despite extensive research on this topic, intervention to improve health literacy is lacking.[3] Some researchers state that when screening for health literacy there is a risk of stigmatization, making the patient feel ashamed for not being able to understand and comprehend health information well enough.[56] Our result showed that 55% (n=390) reported limited functional health literacy (including both inadequate and problematic functional health literacy) and 40% (n=285) had limited communicative and critical health literacy. Our results support the fact that there seems to be a higher prevalence of limited health literacy in patients with severe obesity undergoing bariatric surgery irrespective of country of origin. [24-26]

Despite the growing knowledge of the concept of health literacy there are very few interventional studies in clinical settings.[3] In the future a possible interventional study could use reliable and valid instruments to evaluate the effect of a potential intervention.

Limitations

Despite the strengths of the multicentre design, with high inclusion rates and response rates, the study is not without limitations. The previous psychometric evaluations on these scales are quite sparse, making it difficult to compare results between studies. Therefore, psychometric evaluations need to be ongoing in different contexts to improve external validity on both scales. Both scales used in this study are shorter than other health literacy instruments; however, both scales are considered easy to use and patient friendly in a clinical context. The time required to fill out the forms before surgery might have had on impact on patients' answers. A better alternative might have been to collect data earlier in the preoperative period. According to the literature there is no consensus as to the optimal timing for the measurement of health literacy in a surgical population (pre- or post-operative).[18]

Conclusion

The Swedish functional health literacy scale and the Swedish communicative and critical health literacy scale are valid and reliable instruments to use for patients undergoing bariatric surgery in a Swedish context. Measuring health literacy can be used as a guide for the development of health literacy friendly patient information in patients undergoing bariatric surgery.

For peer review only - http://bmjopen.bmj.com/site/about/guidelines.xhtml

Ethics and dissemination

The study has received approval from the ethical review board in Uppsala, Sweden (Number 2018/256). The study results will be disseminated through peer-reviewed publications and conference presentations to the scientific community and social media.
This is an open-access article distributed under the terms of the Creative Commons Attribution Non-commercial License, which permits use, distribution, and reproduction in any medium, provided the original work is properly cited, and the use is non-commercial and otherwise in compliance with the license.

Authors' contributions

Maria Jaensson: Contributed to design, data collection, analysis and interpretation of data; wrote and critically revised the draft of manuscript; approved the final manuscript.
Erik Stenberg: Contributed to design, data collection, interpretation of data; critically revised manuscript and approved the final manuscript.
Yuli Liang: Performed CFA and interpretation of data; wrote and critically revised the draft of the manuscript and approved the final manuscript.
Ulrica Nilsson: Contributed to design, interpretation of data; critically revised the manuscript and approved the final manuscript.
Karuna Dahlberg: Contributed to design, data collection, interpretation of data; critically revised the manuscript and approved the final manuscript.

Funding statement

This work was supported by Örebro University, grant number ORU 2018/00376 and ORU 2018/01219 and by ALF funding Region Örebro County (OLL-886141, OLL-935386 and OLL-939106) and Bengt Ihre Foundation (no grant number).

Competing interests

None declared

Figure legend

Figure 1. Structural equation model for the Swedish FHL scale. Factor loadings were standardized. FHLa-e represent the questions in the Swedish functional health literacy scale.

Figure 2. Standardized parameter estimates for the 2-factor model of Swedish C&C HL. F1 represents the communicative dimension and F2 represents the critical dimension. CC1-5 represent the questions in the Swedish C & C HL scale.

For peer review only - http://bmjopen.bmj.com/site/about/guidelines.xhtml

REFERENCES

1. Smith JA, Ireland S. Towards equity and health literacy. *Health Promot J Austr*: Official Journal of Australian Association of Health Promotion Professionals. 2020;31(1):3.

2. Rudd R. The evolving concept of Health literacy: New directions for health literacy studies. *J Commun Healthc*. 2015;8(1):7-9.

3. Nutbeam D, Muscat DM. Advancing health literacy interventions. *Health Literacy in Clinical Practice and Public Health: IOS Press*; 2020. p. 115-27.

4. Mårtensson L, Wångdahl J. Scale for functional health literacy. Swedish version. Guidelines for use of the scale. Version 3.1 January 2017. Uppsala university. University of Gothenburg; 2017; 08-02-2021.

5. Javadzade SH, Sharifirad G, Radjati F, Mostafavi F, Reisi M, Hasanzade A. Relationship between health literacy, health status, and healthy behaviors among older adults in Isfahan, Iran. *J Educ Health Promot.* 2012;1.

6. Clouston SA, Manganello JA, Richards M. A life course approach to health literacy: the role of gender, educational attainment and lifetime cognitive capability. *Age Ageing*. 2017; 1;46(3):493-499

7. Almaleh R, Helmy Y, Farhat E, Hasan H, Abdelhafez A. Assessment of health literacy among outpatient clinics attendees at Ain Shams University Hospitals, Egypt: a cross-sectional study. *Public Health.* 2017;151:137-45.

8. Wångdahl J, Lytsy P, Mårtensson L, Westerling R. Health literacy among refugees in Sweden–a cross-sectional study. *BMC Public Health*. 2014;14(1):1-12.

9. Protheroe J, Whittle R, Bartlam B, Estacio EV, Clark L, Kurth J. Health literacy, associated lifestyle and demographic factors in adult population of an English city: a cross-sectional survey. *Health Expect.* 2017;20(1):112-9.

10. Berkman ND, Sheridan SL, Donahue KE, Halpern DJ, Crotty K. Low health literacy and health outcomes: an updated systematic review. *Ann Internal Med*. 2011;155(2):97-107.

11. Halleberg Nyman M, Nilsson U, Dahlberg K, Jaensson M. Association Between Functional Health Literacy and Postoperative Recovery, Health Care Contacts, and Health-Related Quality of Life Among Patients Undergoing Day Surgery: Secondary Analysis of a Randomized Clinical Trial. *JAMA Surg*. 2018;153(8):738-45.

12. Jessup RL, Osborne RH, Beauchamp A, Bourne A, Buchbinder R. Health literacy of recently hospitalised patients: a cross-sectional survey using the Health Literacy Questionnaire (HLQ). *BMC Health Serv Res*. 2017;17(1):52.

13. Haun JN, Patel NR, French DD, Campbell RR, Bradham DD, Lapcevic WA. Association between health literacy and medical care costs in an integrated healthcare system: a regional population based study. *BMC Health Serv Res*. 2015;15:249.

14. Howard DH, Gazmararian J, Parker RM. The impact of low health literacy on the medical costs of Medicare managed care enrollees. *Am J Med*. 2005;118(4):371-7.

15. Mahoney ST, Tawfik-Sexton D, Strassle PD, Farrell TM, Duke MC. Effects of education and health literacy on postoperative hospital visits in bariatric surgery. *J Laparo Adv Surg Tech*.

For peer review only - http://bmjopen.bmj.com/site/about/guidelines.xhtml

2018;28(9):1100-4.

16. Roh YH, Koh YD, Kim JO, Noh JH, Gong HS, Baek GH. Patients With Limited Health Literacy Have Similar Preferences but Different Perceptions in Surgical Decision-making for Carpal Tunnel Release. *Clin Orthop Relat Res*. 2018;476(4):846-51.

17. Håkansson EJ, Holmström IK, Kumlin T, Kaminsky E, Skoglund K, Höglander J, et al. " Same same or different?" A review of reviews of person-centered and patient-centered care. *Patient Educ Couns*. 2019;102(1):3.

18. Chang ME, Baker SJ, Marques ICDS, Liwo AN, Chung SK, Richman JS, et al. Health literacy in surgery. HLRP: *Health Lit Res Pract*. 2020;4(1):e46-e65.

19. Ishikawa H, Takeuchi T, Yano E. Measuring functional, communicative, and critical health literacy among diabetic patients. *Diabetes Care*. 2008;31(5):874-9.

20. Ishikawa H, Kiuchi T. Health literacy and health communication. *Biopsychosoc Med*. 2010;4:18.

21. Wangdahl JM, Martensson LI. Measuring health literacy - the Swedish Functional Health Literacy scale. *Scand J Car Sci*. 2015;29(1):165-72.

22. Wangdahl JM, Martensson LI. The communicative and critical health literacy scale--Swedish version. *Scand J Public Health*. 2014;42(1):25-31.

23. Ishikawa H, Nomura K, Sato M, Yano E. Developing a measure of communicative and critical health literacy: a pilot study of Japanese office workers. *Health Promot Int*. 2008;23(3):269-74.

24. Cayci HM, Erdogdu UE, Demirci H, Ardic A, Topak NY, Taymur İ. Effect of health literacy on help-seeking behavior in morbidly obese patients agreeing to bariatric surgery. *Obes Surg*. 2018;28(3):791-7.

25. Wright JP, Edwards GC, Goggins K, Tiwari V, Maiga A, Moses K, et al. Association of Health Literacy With Postoperative Outcomes in Patients Undergoing Major Abdominal Surgery. *JAMA Surg*. 2018;153(2):137-42.

26. Erdogdu UE, Cayci HM, Tardu A, Demirci H, Kisakol G, Guclu M. Health literacy and weight loss after bariatric surgery. *Obes Surg*. 2019;29(12):3948-53.

27. Wiggins T, Guidozzi N, Welbourn R, Ahmed AR, Markar SR. Association of bariatric surgery with all-cause mortality and incidence of obesity-related disease at a population level: A systematic review and meta-analysis. *PLoS Med*. 2020;17(7):e1003206.

28. Sjöström L. Review of the key results from the Swedish Obese Subjects (SOS) trial–a prospective controlled intervention study of bariatric surgery. *J Intern Med*. 2013;273(3):219-34.

29. Kubik JF, Gill RS, Laffin M, Karmali S. The impact of bariatric surgery on psychological health. *J Obes*. 2013;2013:837989.

30. Madan AK, Tichansky DS. Patients postoperatively forget aspects of preoperative patient education. *Obes Surg*. 2005;15(7):1066-9.

31. Streiner DL, Kottner J. Recommendations for reporting the results of studies of instrument and scale development and testing. *J Adv Nurs*. 2014;70(9):1970-9.
32. Jaensson M, Dahlberg K, Nilsson U, Stenberg E. The impact of self-efficacy and health literacy on outcome after bariatric surgery in Sweden: a protocol for a prospective, longitudinal mixed-methods

For peer review only - http://bmjopen.bmj.com/site/about/guidelines.xhtml

study. *BMJ open*. 2019;9(5):e027272.

33. Sundbom M, Näslund I, Näslund E, Ottosson J. High acquisition rate and internal validity in the Scandinavian Obesity Surgery Registry. *Surg Obes Relat Dis*. 2021;17(3):606-14.

34. Mårtensson L, Wångdahl J. Skala för kommunikativ & kritisk hälsolitteracitet. Svensk version. Handledning för hur du ska använda skalan. version 3.0 Uppsala universitet.Göteborgs universitet; 2017 08-02-2021.

35. Sullivan M, Karlsson J, Ware JE, Jr. The Swedish SF-36 Health Survey--I. Evaluation of data quality, scaling assumptions, reliability and construct validity across general populations in Sweden. *Soc Sci Med*. 1995;41(10):1349-58.

36. Sullivan M, Karlsson J. The Swedish SF-36 Health Survey III. Evaluation of criterion-based validity: results from normative population. *J Clin Epidemiol*. 1998;51(11):1105-13.

37. Karlsson J, Taft C, Sjostrom L, Torgerson JS, Sullivan M. Psychosocial functioning in the obese before and after weight reduction: construct validity and responsiveness of the Obesity-related Problems scale. *Int J Obes Relat Metab Disord*. 2003;27(5):617-30.

38. Mokkink LB, Prinsen CA, Patrick DL, Alonso J, Bouter LM, de Vet HC, et al. COSMIN Study Design checklist for Patient-reported outcome measurement instruments. 2019.

39. Prinsen CA, Vohra S, Rose MR, Boers M, Tugwell P, Clarke M, et al. How to select outcome measurement instruments for outcomes included in a "Core Outcome Set"–a practical guideline. *Trials*. 2016;17(1):449.

40. Terwee CB, Bot SD, de Boer MR, van der Windt DA, Knol DL, Dekker J, et al. Quality criteria were proposed for measurement properties of health status questionnaires. *J Clin Epidemiol*. 2007;60(1):34-42.

41. Vet HCWd, Terwee CB, Mokkink LB, Knol DLr. Measurement in Medicine: Cambridge University Press; 2011.

42. Schober P, Boer C, Schwarte LA. Correlation coefficients: appropriate use and interpretation. *Anesth Analg*. 2018;126(5):1763-8.

43. Zegers CA, Gonzales K, Smith LM, Pullen CH, De Alba A, Fiandt K. The psychometric testing of the functional, communicative, and critical health literacy tool. *Patient Educ Couns*. 2020;103(11):2347-52.

44. Bentler PM. Comparative fit indexes in structural models. Psychol Bull. 1990;107(2):238.

45. Arbuckle J. Amos 17 users' guide (SPSS Ed.). Chicago, IL. 2008.

46. Hu Lt, Bentler PM. Cutoff criteria for fit indexes in covariance structure analysis: Conventional criteria versus new alternatives. *Struct Equ modeling*. 1999;6(1):1-55.

47. Education SCfH. Introduction to higher education 2020 [updated 2020-09-19; cited 2021 08-02]. Available from: https://www.studera.nu/startpage/higher-education-studies/higher-education-in-sweden/introduction-to-higher-education-in-sweden/.

48. Education SCfH. Higher vocational education 2020 [cited 2021 08-02]. Available from: https://www.studera.nu/startpage/road-to-studies/other-ways-of-studying/higher-vocational-education/.

For peer review only - http://bmjopen.bmj.com/site/about/guidelines.xhtml

For peer review only

49. Sweden S. 2021 [cited 2021 08-02]. Available from: https://www.scb.se/en/.

50. Rosseel Y. Lavaan: An R package for structural equation modeling and more. Version 0.5–12 (BETA). *J Stat Soft*. 2012;48(2):1-36.

51. DeCastellarnau A. A classification of response scale characteristics that affect data quality: a literature review. *Quality & quantity*. 2018;52(4):1523-59.

52. Wangdahl JM, Dahlberg K, Jaensson M, Nilsson U. Psychometric validation of Swedish and Arabic versions of two health literacy questionnaires, eHEALS and HLS-EU-Q16, for use in a Swedish context: a study protocol. *BMJ open*. 2019;9(9):e029668.

53. Berens E-M, Vogt D, Messer M, Hurrelmann K, Schaeffer D. Health literacy among different age groups in Germany: results of a cross-sectional survey. *BMC Public Health*. 2016;16(1):1-8.

54. Parnell TA, Stichler JF, Barton AJ, Loan LA, Boyle DK, Allen PE, editors. A concept analysis of health literacy. *Nurs Forum*; 2019: Wiley Online Library.

55. WHO. Health Literacy. The solid facts. In: Kickbusch I, Pelikan J, Apfel F, Tsouros A, editors. Copenhagen. Denmark: World Health Organization; 2013. p. 72.

56. Parikh NS, Parker RM, Nurss JR, Baker DW, Williams MV. Shame and health literacy: the unspoken connection. *Patient Educ Couns*. 1996;27(1):33-9.

For peer review only

For peer review only - http://bmjopen.bmj.com/site/about/guidelines.xhtml

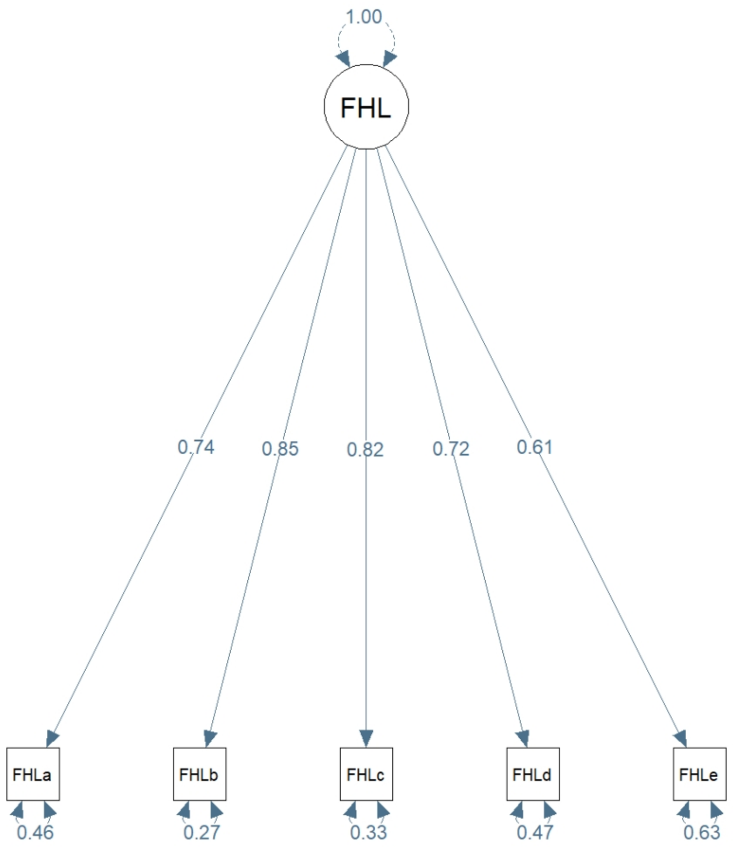

Figure 1. Structural equation model for the Swedish FHL scale. Factor loadings were standardized. FHLa-e represent the questions in the Swedish functional health literacy scale.

101x101mm (300 x 300 DPI)

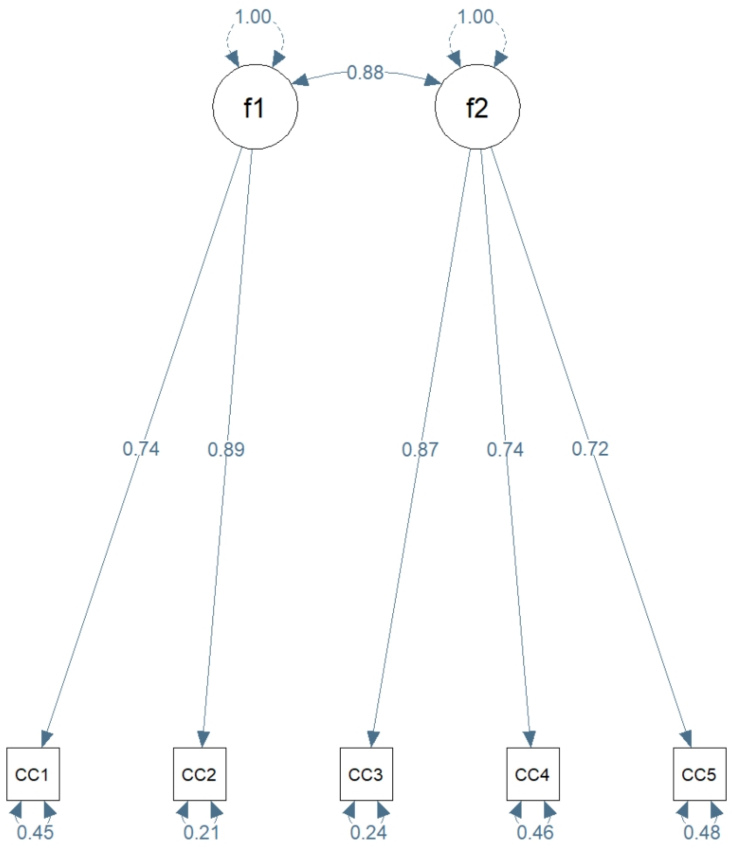

Figure 2. Standardized parameter estimates for the 2-factor model of Swedish C&C HL. F1 represents the communicative dimension and F2 represents the critical dimension. CC1-5 represent the questions in the Swedish C & C HL scale.

101x101mm (300 x 300 DPI)

Reporting checklist for cross sectional study.

Based on the STROBE cross sectional guidelines.

Instructions to authors

Complete this checklist by entering the page numbers from your manuscript where readers will find each of the items listed below.

Your article may not currently address all the items on the checklist. Please modify your text to include the missing information. If you are certain that an item does not apply, please write "n/a" and provide a short explanation.

Upload your completed checklist as an extra file when you submit to a journal.

In your methods section, say that you used the STROBE cross sectionalreporting guidelines, and cite them as:

von Elm E, Altman DG, Egger M, Pocock SJ, Gotzsche PC, Vandenbroucke JP. The Strengthening the Reporting of Observational Studies in Epidemiology (STROBE) Statement: guidelines for reporting observational studies.

For peer review only

		Reporting Item	Page Number
**Title and abstract**			
Title	#1a	Indicate the study’s design with a commonly used term in the title or the abstract	1
Abstract	#1b	Provide in the abstract an informative and balanced summary	2

		of what was done and what was found	
**Introduction**			
Background / rationale	#2	Explain the scientific background and rationale for the investigation being reported	4-5
Objectives	#3	State specific objectives, including any prespecified hypotheses	5
**Methods**			
Study design	#4	Present key elements of study design early in the paper	5
Setting	#5	Describe the setting, locations, and relevant dates, including periods of recruitment, exposure, follow-up, and data collection	5
Eligibility criteria	#6a	Give the eligibility criteria, and the sources and methods of selection of participants.	5
	#7	Clearly define all outcomes, exposures, predictors, potential confounders, and effect modifiers. Give diagnostic criteria, if applicable	5-6
Data sources / measurement	#8	For each variable of interest give sources of data and details of methods of assessment (measurement). Describe comparability of assessment methods if there is more than one group. Give information separately for for exposed and unexposed groups if applicable.	n/a
Bias	#9	Describe any efforts to address potential sources of bias	n/a
Study size	#10	Explain how the study size was arrived at	n/a

For peer review only

Quantitative variables	#11	Explain how quantitative variables were handled in the analyses. If applicable, describe which groupings were chosen, and why	6-7
Statistical methods	#12a	Describe all statistical methods, including those used to control for confounding	6-7
Statistical methods	#12b	Describe any methods used to examine subgroups and interactions	n/a
Statistical methods	#12c	Explain how missing data were addressed	7
Statistical methods	#12d	If applicable, describe analytical methods taking account of sampling strategy	n/a
Statistical methods	#12e	Describe any sensitivity analyses	n/a
**Results**			
Participants	#13a	Report numbers of individuals at each stage of study—eg numbers potentially eligible, examined for eligibility, confirmed eligible, included in the study, completing follow-up, and analysed. Give information separately for for exposed and unexposed groups if applicable.	7
Participants	#13b	Give reasons for non-participation at each stage	7
Participants	#13c	Consider use of a flow diagram	n/a
Descriptive data	#14a	Give characteristics of study participants (eg demographic,	8-9

For peer review only

		clinical, social) and information on exposures and potential confounders. Give information separately for exposed and unexposed groups if applicable.	
Descriptive data	#14b	Indicate number of participants with missing data for each variable of interest	7, 10
Outcome data	#15	Report numbers of outcome events or summary measures. Give information separately for exposed and unexposed groups if applicable.	7-11
Main results	#16a	Give unadjusted estimates and, if applicable, confounder-adjusted estimates and their precision (eg, 95% confidence interval). Make clear which confounders were adjusted for and why they were included	n/a
Main results	#16b	Report category boundaries when continuous variables were categorized	n/a
Main results	#16c	If relevant, consider translating estimates of relative risk into absolute risk for a meaningful time period	n/a
Other analyses	#17	Report other analyses done—e.g., analyses of subgroups and interactions, and sensitivity analyses	n/a

Discussion

Key results	#18	Summarise key results with reference to study objectives	12
Limitations	#19	Discuss limitations of the study, taking into account sources of potential bias or imprecision. Discuss both direction and magnitude of any potential bias.	13

For peer review only

Interpretation	#20	Give a cautious overall interpretation considering objectives, limitations, multiplicity of analyses, results from similar studies, and other relevant evidence.	12-13
Generalisability	#21	Discuss the generalisability (external validity) of the study results	12

Other Information

Funding	#22	Give the source of funding and the role of the funders for the present study and, if applicable, for the original study on which the present article is based	14

None The STROBE checklist is distributed under the terms of the Creative Commons Attribution License CC-BY. This checklist can be completed online using https://www.goodreports.org/, a tool made by the EQUATOR Network in collaboration with Penelope.ai

For peer review only

BMJ Open

Validity and reliability of the Swedish Functional Health Literacy scale and the Swedish Communicative and Critical Health Literacy scale in patients undergoing bariatric surgery in Sweden: a prospective psychometric evaluation study

Journal:	*BMJ Open*
Manuscript ID	bmjopen-2021-056592.R1
Article Type:	Original research
Date Submitted by the Author:	19-Oct-2021
Complete List of Authors:	Jaensson, Maria; Örebro University, Faculty of Medicine and Health, School of Health Sciences<br>Stenberg, Erik; Örebro universitet Fakulteten för medicin och hälsa, Faculty of Medicine and Health<br>Liang, Yuli; Örebro University School of Business, Department of Statistics<br>Nilsson, Ulrica; Karolinska Institutet, Department of Neurobiology, Care Sciences and Society, Karolinska Institutet Perioperative Medicine and Intensive Care; Karolinska Universitetssjukhuset, Perioperative Medicine and Intensive Care<br>Dahlberg, Karuna; Örebro University, Faculty of Medicine and Health, School of Health Sciences
**Primary Subject Heading**:	Surgery
Secondary Subject Heading:	Surgery, Patient-centred medicine
Keywords:	Adult surgery < SURGERY, Health & safety < HEALTH SERVICES ADMINISTRATION & MANAGEMENT, STATISTICS & RESEARCH METHODS

SCHOLARONE™
Manuscripts

I, the Submitting Author has the right to grant and does grant on behalf of all authors of the Work (as defined in the below author licence), an exclusive licence and/or a non-exclusive licence for contributions from authors who are: i) UK Crown employees; ii) where BMJ has agreed a CC-BY licence shall apply, and/or iii) in accordance with the terms applicable for US Federal Government officers or employees acting as part of their official duties; on a worldwide, perpetual, irrevocable, royalty-free basis to BMJ Publishing Group Ltd ("BMJ") its licensees and where the relevant Journal is co-owned by BMJ to the co-owners of the Journal, to publish the Work in this journal and any other BMJ products and to exploit all rights, as set out in our licence.

The Submitting Author accepts and understands that any supply made under these terms is made by BMJ to the Submitting Author unless you are acting as an employee on behalf of your employer or a postgraduate student of an affiliated institution which is paying any applicable article publishing charge ("APC") for Open Access articles. Where the Submitting Author wishes to make the Work available on an Open Access basis (and intends to pay the relevant APC), the terms of reuse of such Open Access shall be governed by a Creative Commons licence – details of these licences and which Creative Commons licence will apply to this Work are set out in our licence referred to above.

Other than as permitted in any relevant BMJ Author's Self Archiving Policies, I confirm this Work has not been accepted for publication elsewhere, is not being considered for publication elsewhere and does not duplicate material already published. I confirm all authors consent to publication of this Work and authorise the granting of this licence.

For peer review only

Validity and reliability of the Swedish Functional Health Literacy scale and the Swedish Communicative and Critical Health Literacy scale in patients undergoing bariatric surgery in Sweden: a prospective psychometric evaluation study

Jaensson M[1], Stenberg E [2], Liang, Y[4], Nilsson U[3], Dahlberg K [1]

1. Faculty of Medicine and Health. School of Health Sciences, Örebro University, Örebro, Sweden
2. Department of Surgery, Faculty of Medicine and Health, Örebro University, Örebro, Sweden
3. Department of Neurobiology, Care Sciences, and Society, Karolinska Institutet and Perioperative Medicine, Karolinska University Hospital, Stockholm, Sweden
4. Department of Statistics, Örebro University School of Business, Örebro, Sweden

Corresponding author: Maria Jaensson, School of Health Sciences, Örebro University, Fakultetsgatan 1, SE- 701 82,ÖREBRO, Sweden
maria.jaensson@oru.se

Word count:3654

For peer review only

ABSTRACT

Objectives

The aim was to psychometrically test and evaluate the Swedish functional health literacy scale and the Swedish communicative and critical health literacy scale in patients undergoing bariatric surgery.

Design

A prospective cross-sectional psychometric study

Setting

Patients from three bariatric centers in Sweden were consecutively included in this study.

Participants

A total of 704 patients undergoing bariatric surgery filled in the questionnaires preoperatively. Inclusion criteria were scheduled for primary bariatric surgery (Roux-en-Y gastric bypass or sleeve gastrectomy) and greater than 17 years, proficiency in Swedish.

Primary and secondary measures

Psychometric outcomes of the Swedish Functional Health Literacy scale and the Swedish Communicative and Critical Health Literacy scale

Results

There was a higher proportion of females (74.4%, n=523) to males (25.6%, n=180). The mean age was 42 years (SD 11.5). Limited functional health literacy and limited communicative and critical health literacy (including both inadequate and problematic health literacy) was reported in 55 % (n=390) and 40 % (n=285), respectively. Cronbach alpha for the Swedish Functional Health Literacy scale was α= 0.86 and for the Swedish Communicative and Critical Health Literacy scale, α=0.87. Construct validity showed weak to negative correlations between the Swedish Functional Health Literacy scale and income, education, and SF-36/RAND36 summary scores. Confirmatory factor analysis showed a one-factor solution for the Swedish Functional Health Literacy scale and a two-factor solution for the Swedish Communicative and Critical Health Literacy scale.

Conclusions

The Swedish Functional Health Literacy scale and the Swedish Communicative and Critical Health Literacy scale are valid and reliable to use for patients undergoing bariatric surgery in a Swedish context. Measuring dimensions of health literacy can be used as a guide for the development of health literacy friendly patient information in patients undergoing bariatric surgery.

For peer review only - http://bmjopen.bmj.com/site/about/guidelines.xhtml

ARTICLE SUMMARY

Strengths and limitations of this study

- This is the first psychometric evaluation in a Swedish sample undergoing bariatric surgery.
- This study contributes to the development of valid and reliable scales to be used in a clinical context.
- This study shows high prevalence of limited health literacy in patients undergoing bariatric surgery.
- The timing of preoperative data collection can be a limitation.
- There are few previous psychometric evaluations, making it difficult to compare results.

For peer review only

For peer review only - http://bmjopen.bmj.com/site/about/guidelines.xhtml

INTRODUCTION

The concept of health literacy has been known for more than 3 decades.[1] Health literacy can be defined as "*a person's ability to assess, understand and use information to maintain or improve one´s health*".[2] Health literacy can be divided into different skills or dimensions. Functional health literacy covers basic skills in understanding and using health information. Communicative and critical health literacy refers to more advanced social and cognitive skills in communication, applying and discriminating between different sources of information.[3, 4] In recent years, research has investigated the consequences of limited health literacy for both the individual and the health care system. Factors such as lower educational level, lower household income, refugees, older age, and sex are associated with limited health literacy.[5-10] Limited health literacy has been reported to be associated with poorer general health, poorer postoperative recovery and increased health care cost.[10-15] For the patient with limited health literacy, it can be difficult to adhere to a medication regimen and to understand health information.[10] Of particular note is research that has shown that a patient with limited health literacy may ask fewer questions when meeting and talking to health care providers.[16] Knowledge of health literacy is therefore important for health care providers to provide equal care for all patients, especially when advocating a person-centered approach.[17]

Scales and instruments can measure health literacy, but there is no scale or instrument that is considered the gold standard. A recent systematic review , found 18 different health literacy instruments used in surgical populations, for example TOFHLA (test of functional health literacy in adults), REALM (rapid estimate of adult literacy in medicine), NVS (newest vital sign), HALS (health and adult literacy survey), or BRIEF (brief health literacy screening tool) [18] This study uses two different scales measuring functional health literacy, namely the Swedish Functional Health Literacy (FHL) scale and the Swedish Communicative and Critical Health Literacy (C & C HL) scale. Both scales were originally developed and revised in Japan and have previously been translated to Swedish and are psychometrically tested and found to be valid and reliable in a Swedish context.[19-22] Exploratory factor analysis by Ishikawa et al. supported a one-factor model for both scales.[19, 23] The scales' psychometric properties have been evaluated in different populations, for instance, general population, patients with diabetes, refugees, patients undergoing day surgery, and outpatient clinics attendees.[7, 8, 11, 19, 21, 22]

The prevalence of limited health literacy in a variety of surgical patients has been shown in a meta-analysis to be greater than a third (34%, interquartile range 16%-50%).[18] Previous research, focusing on patients undergoing bariatric surgery, shows that 30-50 % of this population has limited health literacy.[24-26] While the link between socioeconomic factors and obesity remains complex, obesity appears to be more common for those living under less affluent circumstances in high income countries, why it is likely that socioeconomic indifferences could contribute to the high rates of limited health literacy in this population [27] Bariatric surgery leads to a decrease in morbidity and mortality due to obesity and is the most effective method to reduce weight long term.[28, 29] This surgery requires patients to

For peer review only - http://bmjopen.bmj.com/site/about/guidelines.xhtml

adhere to several health care promotion guidelines to minimize surgical complications, lose excessive weight, and learn healthier behavior.[30] Therefore, patients are carefully prepared before surgery with education, among other things. However, analysis of patients' recall of information shows much information is forgotten or may not have been understood.[31] There is a risk, therefore, that patients with limited health literacy cannot fully adhere to desired health care promotion guidelines pre- and postoperatively with weight loss surgery.

When investigating a phenomenon such as health literacy, it is of utmost importance that the scale or instrument is psychometrically evaluated when used in different contexts, recognizing that scales or instruments can be used to design patient information or education or to facilitate communication between health care provider and patient.[3, 32] This study therefore aims to psychometrically test and evaluate the Swedish FHL scale, and the Swedish C & C HL scale in patients undergoing bariatric surgery.

METHOD

Study design

This psychometric evaluation was a cross-sectional survey conducted between October 2018 and November 2020 consisting of preoperative paper-based self-reported functional and communicative and critical health literacy in patients undergoing bariatric surgery. This study is part of a multicentre prospective, longitudinal mixed-methods study with an embedded design.[33]

Ethics

The project was approved by the Regional Ethical Review Board in Uppsala, Sweden (ref: 2018/256) and followed good clinical practice and ethical principles as outlined by the 1964 Helsinki Declaration and its later amendments. All participants received written and oral information, including voluntary written consent and the option to withdraw at any time.

Sample and settings

Patients from three centers in Sweden were consecutively included in this study. The inclusion criteria were planned primary bariatric surgery (Roux-en-Y gastric bypass or sleeve gastrectomy) and greater than 17 years proficiency in Swedish.

All centers performing bariatric surgical procedures in Sweden report to the national quality and research register (Scandinavian Obesity Surgery Registry [SOReg]).[34] Data in the SOReg are collected at different timepoints: baseline (pre- and intraoperatively) and up to 10 years after surgery. For this psychometric study, cross- sectional data has been merged with data from SOReg collected at baseline before surgery.

Information about this study was given by a surgeon (in one center) and a trained research nurse (in two centers) on a preoperative visit. If the patient agreed to participate, informed written consent was collected on the day of the surgery before filling in the study-specific form.

For peer review only - http://bmjopen.bmj.com/site/about/guidelines.xhtml

Patient and Public Involvement

No patient involved.

Data collection

To measure health literacy, the Swedish FHL scale and the Swedish C & C HL scale were used. Both scales consist of 5 items each. The Swedish FHL scale's first two items focus on visual ability related to the design of the text and its accessibility, the next two on the understanding of words and concepts, the fourth on perseverance in reading, and the last on the need for help in reading and understanding information. Responses are on a 5-point ordinal scale (1=never to 5=always) with lower scores indicating sufficient health literacy. [4]

The first three items in the Swedish C & C HL scale focus on the capacity for collecting, extracting, and understanding information related to health. The last two items focus on the abilities required to judge the reliability of the information and to apply health information to everyday life. Responses are on a 5-point ordinal scale (1=strongly disagree to 5=strongly agree), with higher scores indicating a sufficient health literacy. [21, 22]

Neither scale provides an initial summary score; instead, reported variables are re-categorized to three different levels (inadequate, problematic or sufficient health literacy) or to two categories (not inadequate/sufficient and inadequate) following guidelines instructions for the scales [4, 35]

To evaluate construct and discriminant validity, the following baseline variables were collected from SOReg: patient characteristics (age, gender, weight, BMI and type of surgery) and data from two additional scales, both of which have been found to be valid and reliable for Swedish contexts.[36-38] The first, SF-36/RAND36 (hereafter referred to simply as SF-36), measures health-related quality of life and consists of 36 items grouped into eight scales (bodily pain, emotional role, general health, mental health, physical function, role function, social functioning, and vitality). A higher score indicates better health status (ranging from 0 to 100). SF-36 provides two summary scores: the physical component summary (PCS) and the mental component summary (MCS) reflecting overall physical and mental health status. [36, 37] The second, the obesity-related problem (OP) scale measures the impact of obesity on psychosocial functioning. It consists of eight questions on common OPs . A low score indicates better psychosocial functioning (ranging from 0 to 100).[38]

Psychometric testing and statistical analysis `

The psychometric evaluation was guided by the Consensus-Based Standards for the Selection of Health Measurement Instruments (COSMIN) guidelines.[39] There was no priori sample size calculation for this psychometric evaluation study.

Internal consistency measures the degree of interrelatedness and Cronbach alpha; coefficients ≥0. 7 and ≤0.95 were regarded as acceptable. [40, 41] Inter-item correlation was analyzed to indicate if an item was part of the scale; appropriate values were 0.2 to 0.5.[42]

For peer review only - http://bmjopen.bmj.com/site/about/guidelines.xhtml

Floor and ceiling effects for each item were calculated as percentages of ratings at the lowest or highest level and was taken into account if more than 15% reported the lowest or the highest score.[41]

Construct validity was tested with predefined hypotheses drawn from previous research, namely that those participants with limited functional health literacy have significantly lower educational level, lower income, and lower preoperative summary scores in PCS and MCS in SF-36. [9, 10] When analyzing correlations, the Swedish FHL scale and Swedish C & C HL scale were the dependent variables. To investigate differences between groups (health literacy and educational level and income), the Chi-square-test was used. Correlations were analyzed using the Spearman *rho* correlation test and were interpreted as follows: < 0.1 was considered a negligible correlation, 0.1-0.39 weak correlations, 0.4-0.69 moderate correlations, 0.7-0.89 strong correlations, and >0.89 very strong correlations. [43]

Structural validity was tested with confirmatory factor analysis (CFA).[39] We assumed a 1-factor model with 5 items for the Swedish FHL scale and a 2-factor model for the Swedish C & C HL scale.[44] Before running the CFA, the presence of outliers was investigated. Based on the squared Mahalanobis distance, there were 28 outliers and 29 outliers detected in the questionnaires of Swedish FHL and Swedish C&C HL, respectively. The CFA was conducted after removing the outlier observations. The following model fit criteria were used to evaluate the fit of the hypothetical model comparative fit index (CFI) > 0.9 [45], root mean square error of approximation (RMSEA) < 0.08 [46] and standardized root mean square residual (SRMR) < 0.08.[47]

Discriminant validity was tested with the a priori hypotheses that the OP-scale has weak association (r≤0.39) with both the Swedish FHL and the Swedish C & C HL scale.[42]

Sociodemographic variables and health literacy were analyzed descriptively and presented as number, percent, mean and standard deviation (SD).

Functional health literacy was categorized manually into sufficient, problematic and inadequate by re-categorization using the following transformations: 1and 2 became 1; 3 became 100; and 4 and 5 became 1000. All values were summed and answers >1000 interpreted as inadequate, >100 and <1000 problematic, and <100 sufficient functional health literacy. [4] Communicative and critical health literacy was categorized into sufficient, problematic and inadequate by re-categorization as follows; 1and 2 became 1000; 3 became 100;, and 4 and 5 became 1. All values were summed and totals ≥1000 interpreted as inadequate, >100 and <1000 as problematic, and <100 as sufficient communicative and critical health literacy. [35] Health literacy levels (both scales) were further dichotomized into limited (including inadequate/problematic) vs. sufficient. Missing data was handled by listwise deletion.

The self-reported educational level was mapped to low and high educational level (compulsory school, upper secondary school, and higher vocational training vs. first and second cycle education and third cycle study programs) [48]. Higher vocational training in Sweden lasts from one to two years [49]. The self-reported variable of income was mapped to low and high income (cut-off of 300 000 Swedish krona [SEK]/year, where 1 EUR equals approximately 10.8 SEK [June 2021]) and it was guided by the Statistics Sweden website

For peer review only - http://bmjopen.bmj.com/site/about/guidelines.xhtml

which reports a median income of 337 000 SEK/year (2019) for men and women (20-64 year) [50].

A $p<0.05$ value was considered significant. IBM SPSS Statistics, version 26, was used for the calculations. CFA was performed using the lavaan package, version 0.6-3. [51]

RESULTS

A total of 704 patients gave their consent to participate in the study. Six forms were unit nonresponses and 3 forms had partial responses in the Swedish FHL scale. Seven forms were unit nonresponses and 16 forms had partial responses in the Swedish C & C HL. Missing answers varied between 6 and 16 in each item in both scales. Most missing answers were in item 4 in Swedish C & C HL scale, "*Considering the credibility of the information.*". Response rate for the different scales or instruments were 98.7% (n= 695/704) for the Swedish FHL scale, 96.7% (n=681/704) for the Swedish C & C HL scale, and 72.3% (n=509/704) for the SF-36 and OP scales.

There was a higher proportion of females, 74.4% (n=523) to 25.6% (n=180) males. The mean age was 42 years (SD 11.5), and the mean preoperative weight and BMI were 120.2 kilogram (SD 21.9) and 41.7 (SD 5.7) respectively. In this sample, 55% (n=390) reported limited FHL (including both inadequate and problematic functional health literacy) and 40% (n=285) had limited C & C HL. Most patients reported upper secondary school as their highest level of education (52%, n=367/n=698). Almost twenty-five percent (24.6%) had undergone first, second or third cycle programmes at universities. The most frequent type of surgery was gastric bypass (56.8%, n=400) (Table 1).

Table 1. Sociodemographic variables, health literacy results, and type of surgery

**Variables**	
Sex Female/Male n (%)	523(74.4)/180(25.6)
Age years M(SD)	41.77 (11.4)
Weight (kg) M(SD)[a]	120.27 (21.9)
BMI M(SD)[a]	41.77 (5.7)
**Level of educational n (%)**	
Compulsory school	67 (9.6)
Upper secondary school	367 (52.6)
Other education (*i.e.*, higher vocational training)	92 (13.2)
First and second cycle study programmes	171 (24.5)
Third cycle study programmes	1 (0.1)
**Income (SEK[b]) n (%)**	
<200 000	63 (9.1)
200 000-300 000	97 (13.9)
300 000-400 000	135 (19.4)
400 000-500 000	108 (15.3)
500 000-600 000	89 (12.8)

For peer review only - http://bmjopen.bmj.com/site/about/guidelines.xhtml

>600 000	167 (24)
Unknown	37 (5.3)
**Swedish functional health literacy** (FHL) **n (%)**	
Inadequate	115(16)
Problematic	275(39)
Sufficient	305(43)
**Swedish communicative and critical health literacy** (C & C HL) **n (%)**	
Inadequate	45(6)
Problematic	240(34)
Sufficient	395(56)
**Type of surgery** n (%)	
Gastric bypass (GBP)	398 (56.5)
Sleeve gastrectomy (SG)	294 (41.8)
Missing[c]	10
Other[d]	2 (0.2)

[a]n=698, [b]SEK=Swedish kronor, M-mean, SD-standard deviation, , [c]due to postponed or cancelled surgery, [d] only diagnostic laparoscopy due to unexpected finding during surgery.

Item content and mean (SD) scores for the Swedish FHL scale and the Swedish C & C HL scale are seen in Table 2.

Floor ceiling effect/response distribution

There was a floor and ceiling for all items in both scales (Table 2). The Swedish FHL scale had a floor effect with answers skewed to the left and between 24.6 and 61.4% of the answers to each item being "never." For the Swedish C & C HL scale answers were skewed to the right and questions were answered "correct". The ceiling effect varied between 27.6 and 66.9% for all items (Table 2).

Internal consistency

Cronbach alpha for the Swedish FHL scale was α= 0.86 and for the Swedish C & C HL scale, α=0.87. Inter-item correlations varied between r=0.444 and r=0.702 (Swedish FHL scale) and r=0.434 and r=0.702 (Swedish C & C HL scale).

For peer review only - http://bmjopen.bmj.com/site/about/guidelines.xhtml
For peer review only

For peer review only

Table 2. Item content, response distributions for each item, and mean (SD) and inter-item correlations for the Swedish FHL and the Swedish C & C HL scales

**Swedish functional health literacy (FHL)[a]**	**n**	**n (%) missing**	**Response category n (%)**					**Mean (SD)**
			**1**	**2**	**3**	**4**	**5**	
1. Do you think that it is difficult to read health information because the text is difficult to see (even if you have glasses or contact lenses)?	697	7(1)	349(49.6)	187(26.6)	131(18.6)	25(3.6)	5(0.7)	1.78(0.92)
2. Do you think that it is difficult to understand word or numbers in health information?	697	7(1)	269(38.2)	231(32.8)	164(23.3)	28(4.0)	5(0.7)	1.95(0.91)
3. Do you think that it is difficult to understand the message in health information?	697	7(1)	263(37.4)	258(36.6)	153(21.7)	20(2.8)	3(0.4)	1.91(0.86)
4. Do you think that it takes a long time to read health information?	698	6(0.9)	173(24.6)	216(30.7)	234(33.2)	57(8.1)	18(2.6)	2.33(1.01)
5. Do you ever ask someone else to read and explain health information?	698	6(0.9)	432(61.4)	150(21.3)	76(10.8)	32(4.5)	8(1.1)	1.61(0.93)
**Swedish communicative and critical health literacy (C & C HL)[b]**	**n**	**n (%) missing**	**Response category n (%)**					**Mean (SD)**
			**1**	**2**	**3**	**4**	**5**	
1. Seeking information from various) sources	696	8(1.1)	2(0.3)	4(0.6)	36(5.1)	183(26)	471(66.9)	4.61 (0.63)
2. Extracting relevant information	696	8(1.1)	4(0.6)	17(2.4)	84(11.9)	259(36.8)	332(47.2)	4.29(0.81)
3. Understanding and communicating the information	695	9(1.3)	3(0.4)	7(1.0)	112(15.9)	285(40.5)	288(40.9)	4.23(0.76)
4. Considering the credibility of the information	688	16(2.3)	5(0.7)	19(2.7)	178(25.3)	292(41.5)	194(27.6)	3.95(0.84)
5. Making decisions based on the information	693	11(1.6)	4(0.6)	6(0.9)	121(17.2)	320(45.5)	242(34.4)	4.14(0.77)

Swedish Functional Health Literacy (FHL) scale: 1= never, 2=rarely, 3=sometimes, 4=often, 5=always. Swedish Communicative and Critical Health Literacy (C & C HL) scale: 1= not correct at all, 2=incorrect, 3=partly true, 4= correct, 5=exactly right. [a]English translation of items by Wångdahl et al.[4]
[b]English translation of items by Ishikawa et al.[19]

For peer review only - http://bmjopen.bmj.com/site/about/guidelines.xhtml

Construct validity

Bivariate analyses showed significant associations with limited functional health literacy for the independent variables of income (p=0.01) and educational level (p= 0.02). Limited FHL was associated with lower income and lower educational level. There were negligible to weak negative correlations in all four a priori hypotheses (income, education, MCS and PCS) (Table 3). There was no significant association between education and income with communicative and critical health literacy. When analyzing discriminant validity there was a negligible association between the OP scale and the Swedish FHL scale (*rho* 0.054, p= 0.22) and the C & C HL scale (*rho* 0.046, p= 0.31).

Table 3. Spearman *rho* correlations between the Swedish functional health literacy (FHL) scale and both the demographic characteristics and SF 36

**Variable**	**Value,** ***rho***	***P*** **value**
Education level	-.122**	**0.003**
Income	-.097*	**0.01**
SF-36 MCS	-.251**	**<0.001**
SF-36, PCS	-.103	**0.021**

*Correlation is significant at the 0.05 level (two-tailed),
** Correlation is significant at the 0.001 level (two-tailed)
SF-36, MCS=mental component score and PCS=physical component score

Confirmatory factor analysis

The global fit can be assessed by the following criteria: CFI=0.996, RMSEA=0.043 and SRMR=0.013. The model shown in Figure 1 had a very good model fit which indicates that the one-factor solution is a nice fitting model of the Swedish FHL scale in patients undergoing bariatric surgery. Figure 1 shows the path diagram of the Swedish FHL as well as the factor loadings estimated by the maximum likelihood estimation method. The factor FHL explained almost half or more than half of the variances of items a, d, and e (0.46-0.63 with p-values < 0.001) while less than one-third of the variances of items b and c were explained by the factor FHL (0.27 and 0.33 with p-values < 0.001). The factor loadings, which measured the correlations between a specific item and the FHL factor, ranged from 0.61 to 0.85 (p-values < 0.001).

Insert Figure 1

CFA showed a good fit for a two-factor model with correlation between 2 factors given in Figure 2: CFI = 0.996, RMSEA = 0.047 (90% confidence interval 0.007-0.085) and SRMR = 0.012. The value 0.88 on the curved double-headed arrows is the correlation between the two factors. Comparing this 2-correlated-factor CFA model with the one-factor solution shows that the model in Figure 2 has a better fit.

Insert Figure 2

For peer review only - http://bmjopen.bmj.com/site/about/guidelines.xhtml

DISCUSSION

This study shows that the Swedish FHL scale and the Swedish C & C HL scale are valid and reliable to use in patients undergoing bariatric surgery.

This study represents a sufficient sample size according to COSMIN guidelines [38] and also has high response rates with 695/704 (Swedish FHL scale) and 681/704 (Swedish C & C HL scale) responses.

When analyzing the patients' responses, it was evident that both scales showed a skewed response distribution. This resulted from the large proportion of patients that reported answers on either the lower end (Swedish FHL scale) or the higher end (Swedish C & C HL scale) of the items. As far as we know, this response pattern has not been analysed in previous studies. The reason for this result in our study is not clear. It might be a characteristic of patients undergoing bariatric surgery. It might be a drawback of the construction of both scales.[52] However, the response scales in both the Swedish FHL scale and the Swedish C & C HL scale are categorical rating scales not different from other instruments measuring health literacy. For example, the HLS-EU-Q47 and the HL-EU Q16 have similar 4-point Likert scales with response options on a 4-point scale ranging from 'very difficult' to 'very easy. [53]

One item stood out with the most missing answers (n=16), item 4 in the Swedish C & C HL scale, "*Considering the credibility of the information*". The reason for this is not clear though we did not perform a face or content validity assessment. One explanation might be that the patients found it difficult to value their own skill in knowing if information is credible or not. Responses to specific items are not to be found in previous psychometric evaluations. [20, 22, 23], suggesting that they should be studied further to see if it is an issue in other contexts as well.

Our analysis showed that both scales has good reliability with acceptable Cronbach alpha values of 0.86 (Swedish FHL scale) and 0.87 (Swedish C & C HL scale).[42] This internal consistency is in line with recommended values.[40, 41] Our result is also consistent with previous research on the FHL scale (Cronbach alpha of 0.684) and on the C & C HL (Cronbach alpha 0.86).[19, 23] In addition, inter-item correlations showed acceptable values, although one value of 0.702 indicates that the two items measure almost the same thing, with high inter-item correlation values possibly showing that some items in the scales may be redundant. [42]

Construct validity was confirmed for the Swedish FHL scale by showing an association, although weak to negligible, with income, educational level, and overall poorer health. [9, 25, 54] When interpreting these data, it is important to bear in mind that correlation coefficient association strengths are often interpreted literally, a misinterpretation that is problematic because associations do not show causal relationships.[43] Our results contribute to the growing body of evidence surrounding health literacy's impact on the individual as well as on the health care system.[3]

For peer review only - http://bmjopen.bmj.com/site/about/guidelines.xhtml

When applying CFA to investigate the factor structures for the scales, we obtained a 1-factor model for FHL and a 2-factor model for C & C HL. One item (CC3) "*understanding and communicating the information*" is now loaded on the critical dimension instead of the communicative dimension. This loading might be explained by the wording of this item, because "understand" refers more strongly to critical aspects and people often share information with others after some critical analysis. Moreover, a better model fit was observed when CC3 was included in the critical dimension rather than the communicative dimension. Previous studies have also considered communicative and critical dimensions are two processing dimensions.[3, 55]

The concept of health literacy has been known for decades and its importance to equality acknowledged.[56] Extensive research has shown challenges with limited health literacy not only for the individual but also for health care professionals. Despite extensive research on this topic, intervention to improve health literacy is lacking.[3] Some researchers state that when screening for health literacy there is a risk of stigmatization, making the patient feel ashamed for not being able to understand and comprehend health information well enough. [57] Our result showed that 55% (n=390) reported limited functional health literacy (including both inadequate and problematic functional health literacy) and 40% (n=285) had limited communicative and critical health literacy. Our results support the fact that there seems to be a higher prevalence of limited health literacy in patients with severe obesity undergoing bariatric surgery irrespective of country of origin. [24-26]

Despite the growing knowledge of the concept of health literacy there are very few interventional studies in clinical settings.[3] In the future a possible interventional study could use reliable and valid instruments to evaluate the effect of a potential intervention.

Limitations

Despite the strengths of the multicentre design, with high inclusion rates and response rates, the study is not without limitations. The previous psychometric evaluations on these scales are quite sparse, making it difficult to compare results between studies. Therefore, psychometric evaluations need to be ongoing in different contexts to improve external validity on both scales. Both scales used in this study are shorter than other health literacy instruments; however, both scales are considered easy to use and patient friendly in a clinical context. The time required to fill out the forms before surgery might have had on impact on patients' answers. A better alternative might have been to collect data earlier in the preoperative period. According to the literature there is no consensus as to the optimal timing for the measurement of health literacy in a surgical population (pre- or post-operative).[18]

Our data collection was performed in three centres that can be considered to be medium to high-volume centres operating mostly standard bariatric surgery as well as more advanced bariatric surgery. The samples represent the Swedish (and North European) average patient who undergo bariatric or metabolic surgery. Therefore, our results may not be generalized to other settings and countries. [58]

For peer review only - http://bmjopen.bmj.com/site/about/guidelines.xhtml

Conclusion

The Swedish functional health literacy scale and the Swedish communicative and critical health literacy scale are valid and reliable instruments to use for patients undergoing bariatric surgery in a Swedish context. Measuring health literacy can be used as a guide for the development of health literacy friendly patient information in patients undergoing bariatric surgery.

Data availability statement

No additional data are available

Authors' contributions

Maria Jaensson: Contributed to design, data collection, analysis and interpretation of data; wrote and critically revised the draft of manuscript; approved the final manuscript.
Erik Stenberg: Contributed to design, data collection, interpretation of data; critically revised manuscript and approved the final manuscript.
Yuli Liang: Performed CFA and interpretation of data; wrote and critically revised the draft of the manuscript and approved the final manuscript.
Ulrica Nilsson: Contributed to design, interpretation of data; critically revised the manuscript and approved the final manuscript.
Karuna Dahlberg: Contributed to design, data collection, interpretation of data; critically revised the manuscript and approved the final manuscript.

Funding statement

This work was supported by Örebro University, grant number ORU 2018/00376 and ORU 2018/01219 and by ALF funding Region Örebro County (OLL-886141, OLL-935386 and OLL-939106) and Bengt Ihre Foundation (no grant number).

Competing interests

None declared

Figure legend

Figure 1. Structural equation model for the Swedish FHL scale. Factor loadings were standardized. FHLa-e represent the questions in the Swedish functional health literacy scale.

Figure 2. Standardized parameter estimates for the 2-factor model of Swedish C&C HL. F1 represents the communicative dimension and F2 represents the critical dimension. CC1-5 represent the questions in the Swedish C & C HL scale.

For peer review only - http://bmjopen.bmj.com/site/about/guidelines.xhtml

REFERENCES

1. Smith JA, Ireland S. Towards equity and health literacy. *Health Promot J Austr*: Official Journal of Australian Association of Health Promotion Professionals. 2020;31(1):3.

2. Rudd R. The evolving concept of Health literacy: New directions for health literacy studies. *J Commun Healthc*. 2015;8(1):7-9.

3. Nutbeam D, Muscat DM. Advancing health literacy interventions. *Health Literacy in Clinical Practice and Public Health: IOS Press*; 2020. p. 115-27.

4. Mårtensson L, Wångdahl J. Scale for functional health literacy. Swedish version. Guidelines for use of the scale. Version 3.1 January 2017. Uppsala university. University of Gothenburg; 2017; 08-02-2021.

5. Javadzade SH, Sharifirad G, Radjati F, Mostafavi F, Reisi M, Hasanzade A. Relationship between health literacy, health status, and healthy behaviors among older adults in Isfahan, Iran. *J Educ Health Promot.* 2012;1.

6. Clouston SA, Manganello JA, Richards M. A life course approach to health literacy: the role of gender, educational attainment and lifetime cognitive capability. *Age Ageing*. 2017; 1;46(3):493-499

7. Almaleh R, Helmy Y, Farhat E, Hasan H, Abdelhafez A. Assessment of health literacy among outpatient clinics attendees at Ain Shams University Hospitals, Egypt: a cross-sectional study. *Public Health.* 2017;151:137-45.

8. Wångdahl J, Lytsy P, Mårtensson L, Westerling R. Health literacy among refugees in Sweden–a cross-sectional study. *BMC Public Health*. 2014;14(1):1-12.

9. Protheroe J, Whittle R, Bartlam B, Estacio EV, Clark L, Kurth J. Health literacy, associated lifestyle and demographic factors in adult population of an English city: a cross-sectional survey. *Health Expect.* 2017;20(1):112-9.

10. Berkman ND, Sheridan SL, Donahue KE, Halpern DJ, Crotty K. Low health literacy and health outcomes: an updated systematic review. *Ann Internal Med*. 2011;155(2):97-107.

11. Halleberg Nyman M, Nilsson U, Dahlberg K, Jaensson M. Association Between Functional Health Literacy and Postoperative Recovery, Health Care Contacts, and Health-Related Quality of Life Among Patients Undergoing Day Surgery: Secondary Analysis of a Randomized Clinical Trial. *JAMA Surg*. 2018;153(8):738-45.

12. Jessup RL, Osborne RH, Beauchamp A, Bourne A, Buchbinder R. Health literacy of recently hospitalised patients: a cross-sectional survey using the Health Literacy Questionnaire (HLQ). *BMC Health Serv Res*. 2017;17(1):52.

13. Haun JN, Patel NR, French DD, Campbell RR, Bradham DD, Lapcevic WA. Association between health literacy and medical care costs in an integrated healthcare system: a regional population based study. *BMC Health Serv Res*. 2015;15:249.

14. Howard DH, Gazmararian J, Parker RM. The impact of low health literacy on the medical costs of Medicare managed care enrollees. *Am J Med*. 2005;118(4):371-7.

For peer review only - http://bmjopen.bmj.com/site/about/guidelines.xhtml

15. Mahoney ST, Tawfik-Sexton D, Strassle PD, Farrell TM, Duke MC. Effects of education and health literacy on postoperative hospital visits in bariatric surgery. *J Laparo Adv Surg Tech*. 2018;28(9):1100-4.

16. Roh YH, Koh YD, Kim JO, Noh JH, Gong HS, Baek GH. Patients With Limited Health Literacy Have Similar Preferences but Different Perceptions in Surgical Decision-making for Carpal Tunnel Release. *Clin Orthop Relat Res*. 2018;476(4):846-51.

17. Håkansson EJ, Holmström IK, Kumlin T, Kaminsky E, Skoglund K, Höglander J, et al. " Same same or different?" A review of reviews of person-centered and patient-centered care. *Patient Educ Couns*. 2019;102(1):3.

18. Chang ME, Baker SJ, Marques ICDS, Liwo AN, Chung SK, Richman JS, et al. Health literacy in surgery. HLRP: *Health Lit Res Pract*. 2020;4(1):e46-e65.

19. Ishikawa H, Takeuchi T, Yano E. Measuring functional, communicative, and critical health literacy among diabetic patients. *Diabetes Care*. 2008;31(5):874-9.

20. Ishikawa H, Kiuchi T. Health literacy and health communication. *Biopsychosoc Med*. 2010;4:18.

21. Wangdahl JM, Martensson LI. Measuring health literacy - the Swedish Functional Health Literacy scale. *Scand J Car Sci*. 2015;29(1):165-72.

22. Wangdahl JM, Martensson LI. The communicative and critical health literacy scale--Swedish version. *Scand J Public Health*. 2014;42(1):25-31.

23. Ishikawa H, Nomura K, Sato M, Yano E. Developing a measure of communicative and critical health literacy: a pilot study of Japanese office workers. *Health Promot Int*. 2008;23(3):269-74.

24. Cayci HM, Erdogdu UE, Demirci H, Ardic A, Topak NY, Taymur İ. Effect of health literacy on help-seeking behavior in morbidly obese patients agreeing to bariatric surgery. *Obes Surg*. 2018;28(3):791-7.

25. Wright JP, Edwards GC, Goggins K, Tiwari V, Maiga A, Moses K, et al. Association of Health Literacy With Postoperative Outcomes in Patients Undergoing Major Abdominal Surgery. *JAMA Surg*. 2018;153(2):137-42.

26. Erdogdu UE, Cayci HM, Tardu A, Demirci H, Kisakol G, Guclu M. Health literacy and weight loss after bariatric surgery. *Obes Surg*. 2019;29(12):3948-53.

27. Adams, J. Addressing socioeconomic inequalities in obesity: Democratising access to resources for achieving and maintaining a healthy weight. *PLoS Medicine*. 2020;17(7), e1003243.

28. Wiggins T, Guidozzi N, Welbourn R, Ahmed AR, Markar SR. Association of bariatric surgery with all-cause mortality and incidence of obesity-related disease at a population level: A systematic review and meta-analysis. *PLoS Med*. 2020;17(7):e1003206.

29. Sjöström L. Review of the key results from the Swedish Obese Subjects (SOS) trial–a prospective controlled intervention study of bariatric surgery. *J Intern Med*. 2013;273(3):219-34.

30. Kubik JF, Gill RS, Laffin M, Karmali S. The impact of bariatric surgery on psychological health. *J Obes*. 2013;2013:837989.

For peer review only - http://bmjopen.bmj.com/site/about/guidelines.xhtml

31. Madan AK, Tichansky DS. Patients postoperatively forget aspects of preoperative patient education. *Obes Surg*. 2005;15(7):1066-9.

32. Streiner DL, Kottner J. Recommendations for reporting the results of studies of instrument and scale development and testing. *J Adv Nurs*. 2014;70(9):1970-9.

33. Jaensson M, Dahlberg K, Nilsson U, Stenberg E. The impact of self-efficacy and health literacy on outcome after bariatric surgery in Sweden: a protocol for a prospective, longitudinal mixed-methods study. *BMJ open*. 2019;9(5):e027272.

34. Sundbom M, Näslund I, Näslund E, Ottosson J. High acquisition rate and internal validity in the Scandinavian Obesity Surgery Registry. *Surg Obes Relat Dis*. 2021;17(3):606-14.

35. Mårtensson L, Wångdahl J. Skala för kommunikativ & kritisk hälsolitteracitet. Svensk version. Handledning för hur du ska använda skalan. version 3.0 Uppsala universitet.Göteborgs universitet; 2017 08-02-2021.

36. Sullivan M, Karlsson J, Ware JE, Jr. The Swedish SF-36 Health Survey--I. Evaluation of data quality, scaling assumptions, reliability and construct validity across general populations in Sweden. *Soc Sci Med*. 1995;41(10):1349-58.

37. Sullivan M, Karlsson J. The Swedish SF-36 Health Survey III. Evaluation of criterion-based validity: results from normative population. *J Clin Epidemiol*. 1998;51(11):1105-13.

38. Karlsson J, Taft C, Sjostrom L, Torgerson JS, Sullivan M. Psychosocial functioning in the obese before and after weight reduction: construct validity and responsiveness of the Obesity-related Problems scale. *Int J Obes Relat Metab Disord*. 2003;27(5):617-30.

39. Mokkink LB, Prinsen CA, Patrick DL, Alonso J, Bouter LM, de Vet HC, et al. COSMIN Study Design checklist for Patient-reported outcome measurement instruments. 2019.

40. Prinsen CA, Vohra S, Rose MR, Boers M, Tugwell P, Clarke M, et al. How to select outcome measurement instruments for outcomes included in a "Core Outcome Set"–a practical guideline. *Trials*. 2016;17(1):449.

41. Terwee CB, Bot SD, de Boer MR, van der Windt DA, Knol DL, Dekker J, et al. Quality criteria were proposed for measurement properties of health status questionnaires. *J Clin Epidemiol*. 2007;60(1):34-42.

42. Vet HCWd, Terwee CB, Mokkink LB, Knol DLr. Measurement in Medicine: Cambridge University Press; 2011.

43. Schober P, Boer C, Schwarte LA. Correlation coefficients: appropriate use and interpretation. *Anesth Analg*. 2018;126(5):1763-8.

44. Zegers CA, Gonzales K, Smith LM, Pullen CH, De Alba A, Fiandt K. The psychometric testing of the functional, communicative, and critical health literacy tool. *Patient Educ Couns*. 2020;103(11):2347-52.

45. Bentler PM. Comparative fit indexes in structural models. Psychol Bull. 1990;107(2):238.

46. Arbuckle J. Amos 17 users' guide (SPSS Ed.). Chicago, IL. 2008.

For peer review only - http://bmjopen.bmj.com/site/about/guidelines.xhtml

47. Hu Lt, Bentler PM. Cutoff criteria for fit indexes in covariance structure analysis: Conventional criteria versus new alternatives. *Struct Equ modeling*. 1999;6(1):1-55.

48. Education SCfH. Introduction to higher education 2020 [updated 2020-09-19; cited 2021 08-02]. Available from: https://www.studera.nu/startpage/higher-education-studies/higher-education-in-sweden/introduction-to-higher-education-in-sweden/.

49. Education SCfH. Higher vocational education 2020 [cited 2021 08-02]. Available from: https://www.studera.nu/startpage/road-to-studies/other-ways-of-studying/higher-vocational-education/.

50. Statistics Sweden . 2021 [cited 2021 08-02]. Available from: https://www.scb.se/en/.

51. Rosseel Y. Lavaan: An R package for structural equation modeling and more. Version 0.5–12 (BETA). *J Stat Soft*. 2012;48(2):1-36.

52. DeCastellarnau A. A classification of response scale characteristics that affect data quality: a literature review. *Quality & quantity*. 2018;52(4):1523-59.

53. Wangdahl JM, Dahlberg K, Jaensson M, Nilsson U. Psychometric validation of Swedish and Arabic versions of two health literacy questionnaires, eHEALS and HLS-EU-Q16, for use in a Swedish context: a study protocol. *BMJ open*. 2019;9(9):e029668.

54. Berens E-M, Vogt D, Messer M, Hurrelmann K, Schaeffer D. Health literacy among different age groups in Germany: results of a cross-sectional survey. *BMC Public Health*. 2016;16(1):1-8.

55. Parnell TA, Stichler JF, Barton AJ, Loan LA, Boyle DK, Allen PE, editors. A concept analysis of health literacy. *Nurs Forum*; 2019: Wiley Online Library.

56. WHO. Health Literacy. The solid facts. In: Kickbusch I, Pelikan J, Apfel F, Tsouros A, editors. Copenhagen. Denmark: World Health Organization; 2013. p. 72.

57. Parikh NS, Parker RM, Nurss JR, Baker DW, Williams MV. Shame and health literacy: the unspoken connection. *Patient Educ Couns*. 1996;27(1):33-9.

58. Welbourn, R., Hollyman, M., Kinsman, R., Dixon, J., Liem, R., Ottosson, J., ... & Himpens, J. (2019). Bariatric surgery worldwide: baseline demographic description and one-year outcomes from the fourth IFSO global registry report 2018. Obesity surgery, 29(3), 782-795.

For peer review only

For peer review only - http://bmjopen.bmj.com/site/about/guidelines.xhtml

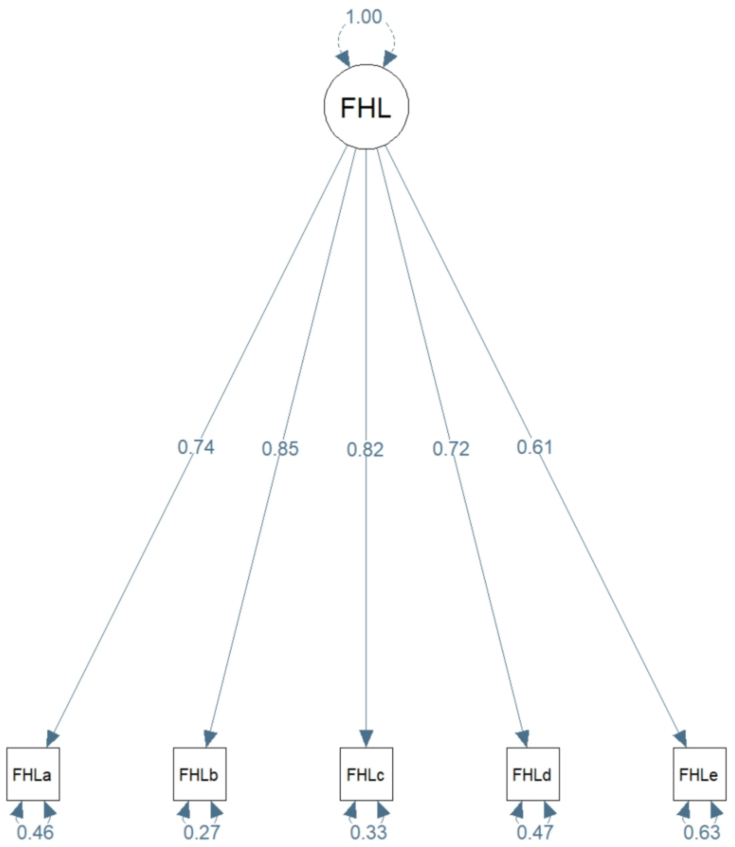

Figure 1. Structural equation model for the Swedish FHL scale. Factor loadings were standardized. FHLa-e represent the questions in the Swedish functional health literacy scale.

101x101mm (300 x 300 DPI)

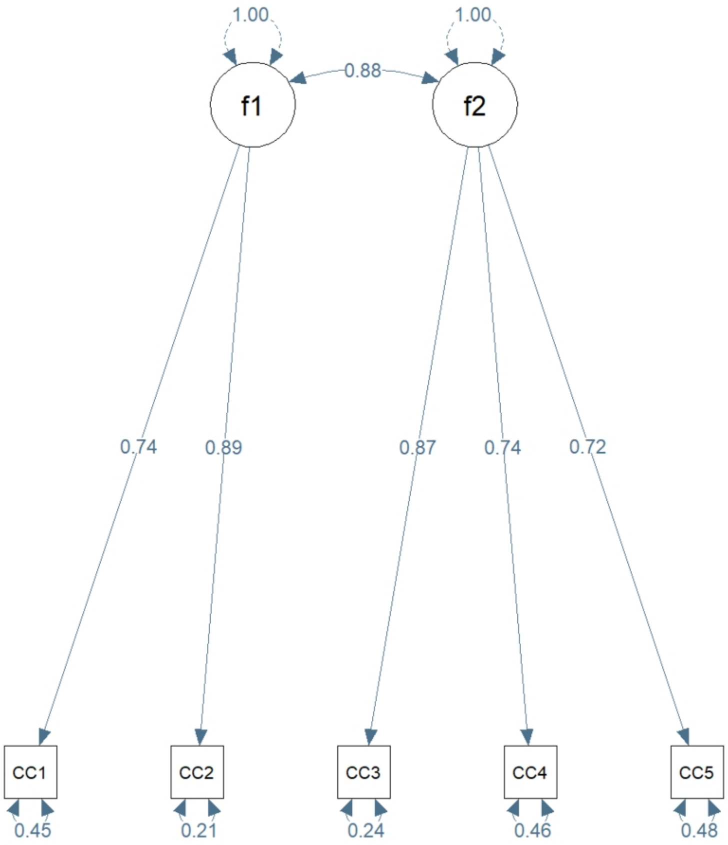

Figure 2. Standardized parameter estimates for the 2-factor model of Swedish C&C HL. F1 represents the communicative dimension and F2 represents the critical dimension. CC1-5 represent the questions in the Swedish C & C HL scale.

101x101mm (300 x 300 DPI)

Reporting checklist for cross sectional study.

Based on the STROBE cross sectional guidelines.

Instructions to authors

Complete this checklist by entering the page numbers from your manuscript where readers will find each of the items listed below.

Your article may not currently address all the items on the checklist. Please modify your text to include the missing information. If you are certain that an item does not apply, please write "n/a" and provide a short explanation.

Upload your completed checklist as an extra file when you submit to a journal.

In your methods section, say that you used the STROBE cross sectionalreporting guidelines, and cite them as:

von Elm E, Altman DG, Egger M, Pocock SJ, Gotzsche PC, Vandenbroucke JP. The Strengthening the Reporting of Observational Studies in Epidemiology (STROBE) Statement: guidelines for reporting observational studies.

		Reporting Item	Page Number
**Title and abstract**			
Title	#1a	Indicate the study's design with a commonly used term in the title or the abstract	1
Abstract	#1b	Provide in the abstract an informative and balanced summary	2

For peer review only

		of what was done and what was found	
**Introduction**			
Background / rationale	#2	Explain the scientific background and rationale for the investigation being reported	4-5
Objectives	#3	State specific objectives, including any prespecified hypotheses	5
**Methods**			
Study design	#4	Present key elements of study design early in the paper	5
Setting	#5	Describe the setting, locations, and relevant dates, including periods of recruitment, exposure, follow-up, and data collection	5
Eligibility criteria	#6a	Give the eligibility criteria, and the sources and methods of selection of participants.	5
	#7	Clearly define all outcomes, exposures, predictors, potential confounders, and effect modifiers. Give diagnostic criteria, if applicable	5-6
Data sources / measurement	#8	For each variable of interest give sources of data and details of methods of assessment (measurement). Describe comparability of assessment methods if there is more than one group. Give information separately for for exposed and unexposed groups if applicable.	n/a
Bias	#9	Describe any efforts to address potential sources of bias	n/a
Study size	#10	Explain how the study size was arrived at	n/a

For peer review only

Quantitative variables	#11	Explain how quantitative variables were handled in the analyses. If applicable, describe which groupings were chosen, and why	6-7
Statistical methods	#12a	Describe all statistical methods, including those used to control for confounding	6-7
Statistical methods	#12b	Describe any methods used to examine subgroups and interactions	n/a
Statistical methods	#12c	Explain how missing data were addressed	7
Statistical methods	#12d	If applicable, describe analytical methods taking account of sampling strategy	n/a
Statistical methods	#12e	Describe any sensitivity analyses	n/a
**Results**			
Participants	#13a	Report numbers of individuals at each stage of study—eg numbers potentially eligible, examined for eligibility, confirmed eligible, included in the study, completing follow-up, and analysed. Give information separately for for exposed and unexposed groups if applicable.	7
Participants	#13b	Give reasons for non-participation at each stage	7
Participants	#13c	Consider use of a flow diagram	n/a
Descriptive data	#14a	Give characteristics of study participants (eg demographic,	8-9

For peer review only

		clinical, social) and information on exposures and potential confounders. Give information separately for exposed and unexposed groups if applicable.	
Descriptive data	#14b	Indicate number of participants with missing data for each variable of interest	7, 10
Outcome data	#15	Report numbers of outcome events or summary measures. Give information separately for exposed and unexposed groups if applicable.	7-11
Main results	#16a	Give unadjusted estimates and, if applicable, confounder-adjusted estimates and their precision (eg, 95% confidence interval). Make clear which confounders were adjusted for and why they were included	n/a
Main results	#16b	Report category boundaries when continuous variables were categorized	n/a
Main results	#16c	If relevant, consider translating estimates of relative risk into absolute risk for a meaningful time period	n/a
Other analyses	#17	Report other analyses done—e.g., analyses of subgroups and interactions, and sensitivity analyses	n/a
**Discussion**			
Key results	#18	Summarise key results with reference to study objectives	12
Limitations	#19	Discuss limitations of the study, taking into account sources of potential bias or imprecision. Discuss both direction and magnitude of any potential bias.	13

For peer review only

Interpretation	#20	Give a cautious overall interpretation considering objectives, limitations, multiplicity of analyses, results from similar studies, and other relevant evidence.	12-13
Generalisability	#21	Discuss the generalisability (external validity) of the study results	12

Other Information

Funding	#22	Give the source of funding and the role of the funders for the present study and, if applicable, for the original study on which the present article is based	14

None The STROBE checklist is distributed under the terms of the Creative Commons Attribution License CC-BY. This checklist can be completed online using https://www.goodreports.org/, a tool made by the EQUATOR Network in collaboration with Penelope.ai

For peer review only
